# Widening the Spectrum of Disease Expression due to Heterozygous Variants in *EFEMP1*

**DOI:** 10.1001/jamaophthalmol.2026.3828

**Published:** 2026-09-10

**Authors:** Chloe M. Stanton, Georg Ansari, Kristina Pfau, Theresa Lipsky, Mihail Halachev, Lukas Gerasimavicius, Camilla Drake, Joseph A. Marsh, Caroline Hayward, Alexander Sumaroka, Bohdan Kousal, Petra Lišková, Mathieu Quinodoz, Carlo Rivolta, Tomas S. Aleman, Artur V. Cideciyan, Maximilian Pfau

**Affiliations:** 1 Centre for Inflammation Research, Institute for Regeneration and Repair, University of Edinburgh, Edinburgh, United Kingdom; 2 Institute of Genetics and Cancer, University of Edinburgh, Edinburgh, United Kingdom; 3 Department of Ophthalmology, University of Basel, Basel, Switzerland; 4 Department of Ophthalmology, University of Bonn, Bonn, Germany; 5 Center for Hereditary Retinal Degenerations, Scheie Eye Institute, Department of Ophthalmology, Perelman School of Medicine, University of Pennsylvania, Philadelphia; 6 Department of Ophthalmology, First Faculty of Medicine, Charles University and General University Hospital in Prague, Prague, Czech Republic; 7 Department of Paediatrics and Inherited Metabolic Disorders, First Faculty of Medicine, Charles University and General University Hospital in Prague, Prague, Czech Republic; 8 Institute of Molecular and Clinical Ophthalmology Basel, Basel, Switzerland

## Abstract

**Importance:**

Doyne honeycomb retinal dystrophy or malattia leventinese (DHRD/ML) is an autosomal dominant retinal disorder caused by a single recurrent p.Arg345Trp (NM_001039348.3:c.1033C>T) variant in *EFEMP1* and characterized by early-onset, nearly confluent, large central retinal drusen. Other variants in *EFEMP1* have not been reported to cause retinal disease.

**Objective:**

To define the topography of the anatomical and functional phenotype of another variant in *EFEMP1*, p.Arg140Trp (c.418C>T), associated with retinal degeneration.

**Design, Setting, and Participants:**

This multi-institutional case series was conducted at 3 inherited retinal disease clinics (Philadelphia, Pennsylvania; Basel, Switzerland; and Prague, Czech Republic) from October 1998 to May 2026. Participants included 3 unrelated families with the p.Arg140Trp variant and 1 comparator family with canonical DHRD/ML caused by the p.Arg345Trp variant. Analyses were conducted from November 2019 to May 2026.

**Exposure:**

Heterozygosity for pathogenic *EFEMP1* variants.

**Main Outcomes and Measures:**

Color fundus photography and optical coherence tomography, light- and dark-adapted chromatic perimetry, and rod-mediated dark adaptation kinetics.

**Results:**

The *EFEMP1* p.Arg140Trp variant segregated with disease in all 3 families. In family 1, the index case developed nyctalopia in the sixth decade with progressive field loss and abnormal ERG. Among 5 at-risk relatives (ages 50 to 58 years), 4 heterozygous carriers had delayed rod-mediated dark adaptation kinetics despite best-corrected visual acuity of 20/20 OU and normal fundus appearance; the noncarrier had normal imaging and function. Additional heterozygous carriers from families 2 (age 42 years; symptom onset at 40 years) and 3 (age 69 years; symptom onset at 55 years) had nyctalopia and peripheral lobular chorioretinal atrophy initially labeled gyrate atrophy-like or choroideremia-like. Topographically matched testing in temporal retina without visible atrophy showed severe rod dysfunction and markedly delayed dark adaptation at 15°, 30°, and 46° with increasing severity toward the periphery. For comparison, the patient with DHRD/ML exhibited normal light- and dark-adapted visual function outside the central 10° and an increasing delay in rod recovery kinetics toward the fovea.

**Conclusions and Relevance:**

This study found that the *EFEMP1* p.Arg140Trp variant was associated with a late-onset, predominantly peripheral retinal degeneration with features overlapping the late-onset retinal degeneration phenotype and was distinct from canonical DHRD/ML; it spares relatively central macular structure and function while preferentially affecting the periphery. Functional evidence suggests preferential rod involvement, with rod dysfunction observed at retinal loci without detectable outer nuclear layer loss.

## Key Points

**Question:**

What is the spectrum of phenotype caused by *EFEMP1* variants?

**Findings:**

In this case series of 3 unrelated families, *EFEMP1* p.Arg140Trp segregated with a late-onset, predominantly peripheral retinal degeneration, and heterozygous carriers showed severe rod dysfunction with markedly delayed dark adaptation kinetics at retinal loci without clinically visible atrophy, while central acuity, structure, and L/M-cone function remained normal.

**Meaning:**

This study found that *EFEMP1* p.Arg140Trp causes a late-onset, predominantly peripheral retinal degeneration distinct from canonical Doyne honeycomb retinal dystrophy or malattia leventinese (DHRD/ML), whereas published and current results in DHRD/ML caused by *EFEMP1* p.Arg345Trp, show a central predilection with peripheral preservation, suggesting that early rod dysfunction preceding outer nuclear layer loss provides a potential intervention window.

## Teaser

This case series examines the anatomical and functional phenotype of a variant in *EFEMP1*, p.Arg140Trp, and its associations with retinal degeneration.

## Introduction

Most patients with inherited retinal degenerations (IRDs) become symptomatic between childhood and early adulthood. Exceptions are a group of late-onset IRDs where symptoms appear in the fifth decade or later and progress over subsequent decades. A well-described member of this group is late-onset retinal degeneration (L-ORD).^1^ Early disease manifestations of L-ORD in midlife include delayed dark adaptation and subtle yellow-white fundus deposits, with later development of lobular chorioretinal atrophy.^2^ Histopathologic studies of affected donor eyes revealed thick, periodic acid–Schiff-positive, lipid-rich sub–retinal pigment epithelium (RPE) deposits, implicating altered outer retinal–Bruch membrane interactions in disease pathogenesis.^1^

With the advent of molecular genetic testing, L-ORD was linked in multiple families to a recurrent *C1QTNF5* p.Ser163Arg variant^3^; additional pathogenic variants in *C1QTNF5* were subsequently identified.^4^ However, one family originally diagnosed as having a clinical and histological phenotype consistent with L-ORD^5^ lacked detectable pathogenic variants in *C1QTNF5*.^2^ In retrospect, this family exhibited some features atypical for L-ORD. Sub-RPE deposits were thinnest in the central macula, where photoreceptors were relatively preserved, and dark adaptation delays were more pronounced in the peripheral retina than centrally in early disease.^5^ Despite the shared features of late-onset disease with thick sub-RPE deposits, findings in this family contrasted with the canonical *C1QTNF5*-associated L-ORD phenotype.^2^

In this multi-institutional case series, we reexamined this family and evaluated 2 additional families with this phenotype, establishing a variant in *EFEMP1* (NM_001039348.3:c.418C>T; p.Arg140Trp) as the underlying molecular cause of this distinct retinal degeneration. To delineate shared and distinguishing phenotypic features, we also evaluated an additional family with Doyne honeycomb retinal dystrophy and malattia leventinese (DHRD/ML) caused by the recurrent *EFEMP1* c.1033C>T (p.Arg345Trp) variant.

## Methods

In this case series, patients were evaluated from October 1998 to May 2026 at 3 specialized IRD clinics using standardized visual function testing and multimodal retinal imaging. All participants provided written informed consent per the Declaration of Helsinki;^6^ study protocols were approved by local ethics committees at each site. The examination included best-corrected visual acuity testing, a slitlamp examination, funduscopy, and color fundus photographs. This case series was reported in accordance with the guidelines proposed by Kempen.^7^

Three unrelated families (families 1-3) harboring the *EFEMP1* p.Arg140Trp variant were studied together with a comparator family (family 4) carrying the canonical DHRD/ML-associated *EFEMP1* p.Arg345Trp variant. The workup combined cross-sectional examination with longitudinal reevaluation where available; ages at first and last examination are listed in the eTable in Supplement 1. Sex was self-reported.

### Retinal Imaging

At site 1 (Philadelphia, Pennsylvania), retinal structure was assessed using time-domain optical coherence tomography (OCT 1 module [Carl Zeiss Meditec]). Scans were acquired along the horizontal and vertical meridians. Individual A-scans were aligned using a dynamic cross-correlation algorithm and laterally smoothed to improve the signal to noise ratio. At site 2 (Basel, Switzerland) and site 3 (Prague, Czech Republic), spectral-domain OCT (SD-OCT) was performed using a Spectralis OCT 2 module system (Heidelberg Engineering).

### Dark Adaptometry

At site 1, rod-mediated dark adaptation was measured following an estimated 97% rhodopsin bleach, using Goldmann size V (1.7° diameter) blue (500 nm) and red (650 nm) test stimuli of 200 milliseconds in duration. Sensitivity recovery was assessed at retinal loci corresponding to 4° (nasal retina), 12° (inferior retina), and 30° (nasal retina).^5^ At site 2, dark adaptometry was measured following a 59% rhodopsin bleach at retinal eccentricities of 8°, 15°, 30°, and 46°, all located in the temporal retina (nasal visual field), with blue (500 nm) and red (647 nm) stimuli (Goldmann size V; 200 milliseconds), using the MonCvONE perimeter (Metrovision).^8^

### Perimetry

At site 2, light-adapted and dark-adapted 2-color static perimetry was also performed using the Perimetry for Inherited Retinal Degeneration (PERIRD) Consortium Protocol, implemented on the MonCvONE platform (Metrovision), enabling separation of L/M-cone–, S-cone–, and rod-mediated visual function across retinal eccentricities. Briefly, the PERIRD protocol comprises 5 stimulus conditions presented along the vertical meridian: (1) light-adapted white-on-white standard automated perimetry, (2) light-adapted blue-on-yellow testing isolating S-cone-mediated sensitivity, (3) light-adapted red-on–dim blue testing isolating L/M-cone-mediated sensitivity, and (4-5) dark-adapted 2-color (blue and red) testing. Stimuli were Goldmann size V (1.7° diameter) and were 200 milliseconds in duration; sensitivity was compared with site-specific normative prediction intervals derived from healthy controls.^9^

### Genetic Testing

Genetic analysis in family 1 used whole-genome sequencing in selected affected and unaffected relatives, followed by panel-based variant filtering and segregation confirmation by polymerase chain reaction and Sanger sequencing with *Hpa*II digest. Functional support for the candidate variant included in silico pathogenicity and structure prediction using the Ensembl Variant Effect Predictor^10^ software version 115 (European Molecular Biology Laboratory, European Bioinformatics Institute) with the integrated tools SIFT (sorting intolerant from tolerant), PolyPhen (polymorphism phenotyping), ClinPred (clinical prediction), REVEL (rare exome variant ensemble learner), AlphaMissense, CADD (combined annotation dependent depletion), BLOSUM62 (Blocks substitution matrix 62), and EVE (evolutionary model of variant effect); in silico mutagenesis and interface modeling using AlphaFold 3 (version 3.0.1 [Google DeepMind and Isomorphic Labs]) and Rosetta version 2021.16, specific build id 2021.16.61629 for Linux (Rosetta Commons) software; and in vitro expression studies of wild-type and variant EFEMP1 (p.Arg140Trp and p.Arg345Trp) in HEK293T cells (full details in the eMethods in Supplement 1). Variant effects on intracellular abundance, multimerization, and secretion were assessed by reducing and nonreducing immunoblotting with densitometric quantification across 3 independent experiments (eMethods in Supplement 1). For sites 2 and 3, whole-exome sequencing and data analysis were performed according to previously specified protocols.^11,12^

### Statistical Analysis

Analyses were conducted from February 2026 to May 2026. Statistical tests included unpaired 2-tailed *t* tests to compare means, with a Welch correction if SDs for each group were not equal, and were conducted with GraphPad Prism for Windows, software version 10.6.1 (GraphPad Software). Significance was defined as *P* < .05.

## Results

### Patient Characteristics

Clinical data were analyzed from 3 unrelated families carrying the same *EFEMP1* p.Arg140Trp variant (pedigrees in eFigure 1 in Supplement 1, genetic testing in eFigure 2 in Supplement 1, and clinical summary in the eTable in Supplement 1), including reanalysis of the previously reported family 1 across 3 generations,^5^ and additional affected individuals from Switzerland (family 2) and the Czech Republic (family 3).

In family 1, the variant segregated with clinically apparent disease or dysfunction across multiple members (eFigure 1 in Supplement 1); the index case (II-1) presented with late-onset night blindness in the sixth decade, progressive visual field loss, abnormal electroretinograms, and characteristic postmortem histopathology previously reported by Milam et al;^5^ no new histopathological material from the index case or from additional carriers was available for the present study. Among 5 individuals from generation III examined at ages 50 to 58 years, 4 heterozygous carriers showed abnormal rod-mediated dark adaptation kinetics despite normal visual acuity (20/20 OU), normal fundus appearance, and normal full-field electroretinogram, whereas the single noncarrier had normal dark adaptation kinetics and normal visual function. Although the present analysis is cross-sectional, longitudinal data were available for 2 carriers in family 1: patient III-1 (re-examined at 50 and 57 years; a 7-year interval) and patient III-5 (re-examined at 44 and 50 years; a 6-year interval). All other participants were assessed at a single time point.

**Figure 1.**
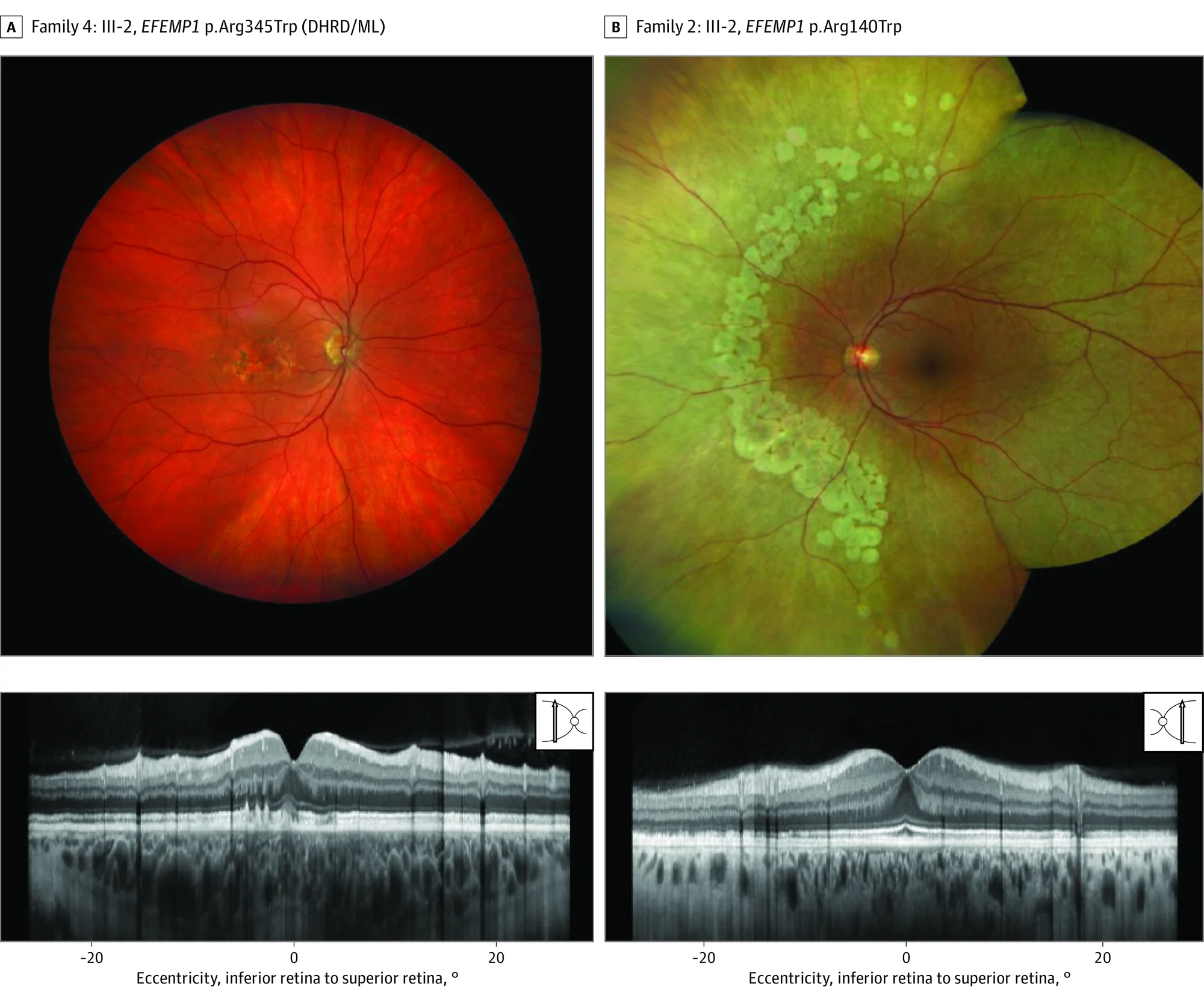
Phenotype of Malattia Leventinese Compared With *EFEMP1* p.Arg140Trp Retinal Degeneration A, A color fundus photograph of a patient with malattia leventinese, demonstrating the pathognomonic phenotype of central drusen-like deposits with associated hyperpigmentary changes. Corresponding spectral-domain optical coherence tomography (OCT) along the vertical meridian shows preservation of overall retinal lamination outside the central macula. B, A fundus photograph of family 2, individual III-2, showing lobular chorioretinal atrophy in the nasal retina at approximately 30° to 45° eccentricity, with subtle yellowish deposits temporally at similar eccentricities. OCT reveals retinal pigment epithelium abnormalities and discontinuity of the ellipsoid zone in affected regions.

Heterozygous carriers from family 2 (examined at age 42 years; symptom onset at 40 years) and family 3 (examined at age 69 years; symptom onset at 55 years) demonstrated nyctalopia with abnormal rod function and peripheral chorioretinal phenotypes diagnosed as gyrate atrophy–like or choroideremia-like fundus appearance (color fundus photographs of family 2, patient III-2 shown in Figure 1; multimodal imaging of family 3, patient II-3 shown in eFigure 3 in Supplement 1). In families 2 and 3, no other first-degree relatives were available for clinical examination. Family history revealed no symptomatic disease in other relatives.

Across families, disease expression was characterized by rod dysfunction preceding widespread structural degeneration, variable peripheral chorioretinal atrophy, and preserved cone-mediated central vision into mid to late adulthood. Clinically, no macular or extramacular choroidal neovascularization (CNV) was detected by multimodal imaging in any examined p.Arg140Trp carrier across families 1 to 3 at the time of examination. However, histopathology of the single eye donor revealed (subclinical) CNV, with new vessels arising from the choriocapillaris and extending through breaks in the elastin layer of the Bruch membrane.^5^

### Structural and Functional Phenotype

**Figure 2.**
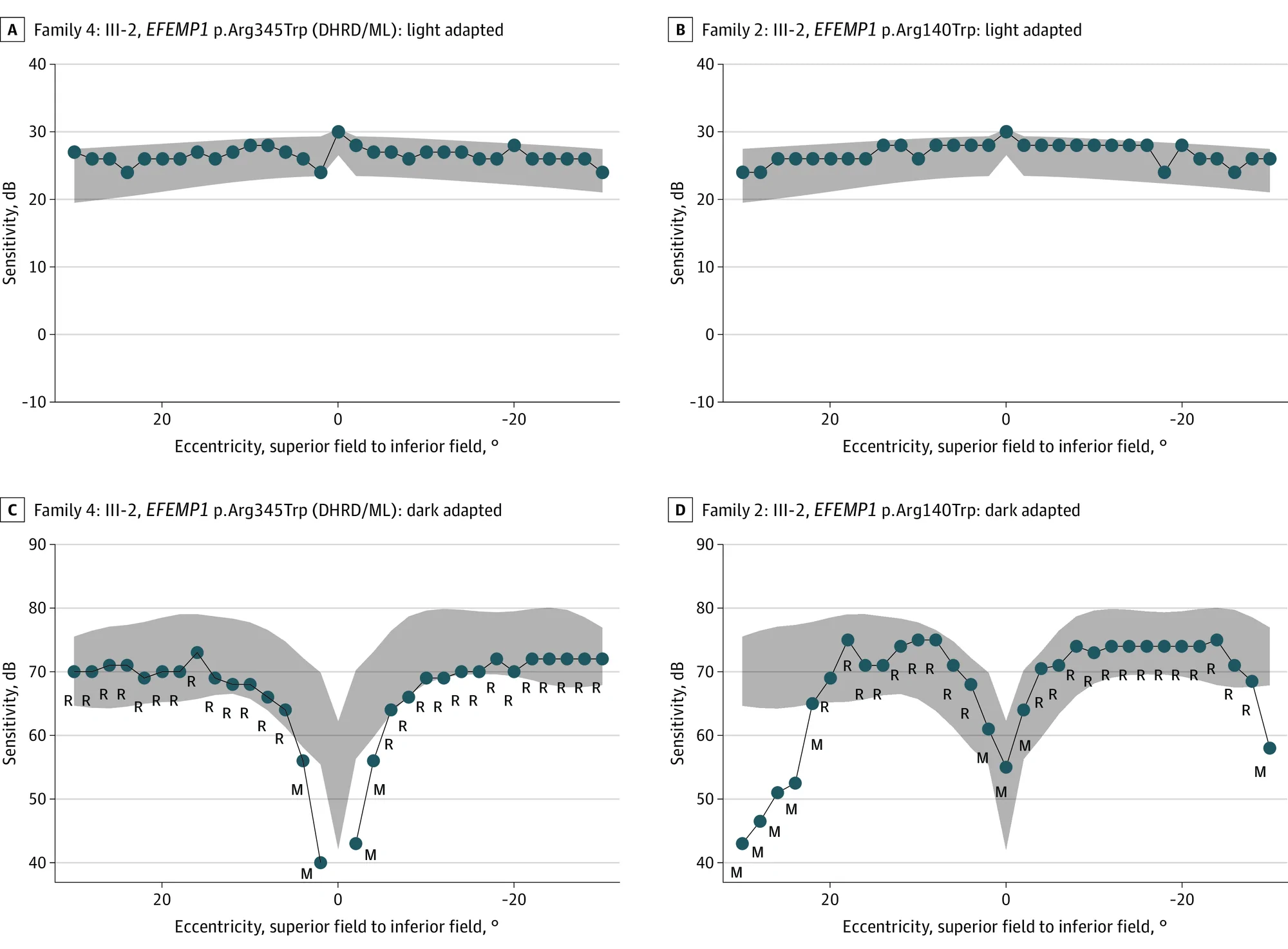
Line Graphs of Static Perimetry in Malattia Leventinese Compared With *EFEMP1* p.Arg140Trp Retinal Degeneration Static perimetry included light-adapted white-on-white and dark-adapted 2-color testing. Family 4, individual III-2 (Doyne honeycomb retinal dystrophy and malattia leventinese [DHRD/ML]) shows near-normal cone and rod sensitivity outside the central 10° (diameter). Family 2, individual III-2 shows nominally greater rod-mediated sensitivity loss on dark-adapted testing, with a peak near 20° in the superior visual field (inferior retina). However, small torsional differences in eye position or head tilt could contribute to the apparent rod-cone dissociation because these locations are close to the atrophic border. R indicates rod mediation of dark-adapted blue and red stimulus; M, mixed mediation of the dark-adapted blue (rod) and red (cone) stimulus.

**Figure 3.**
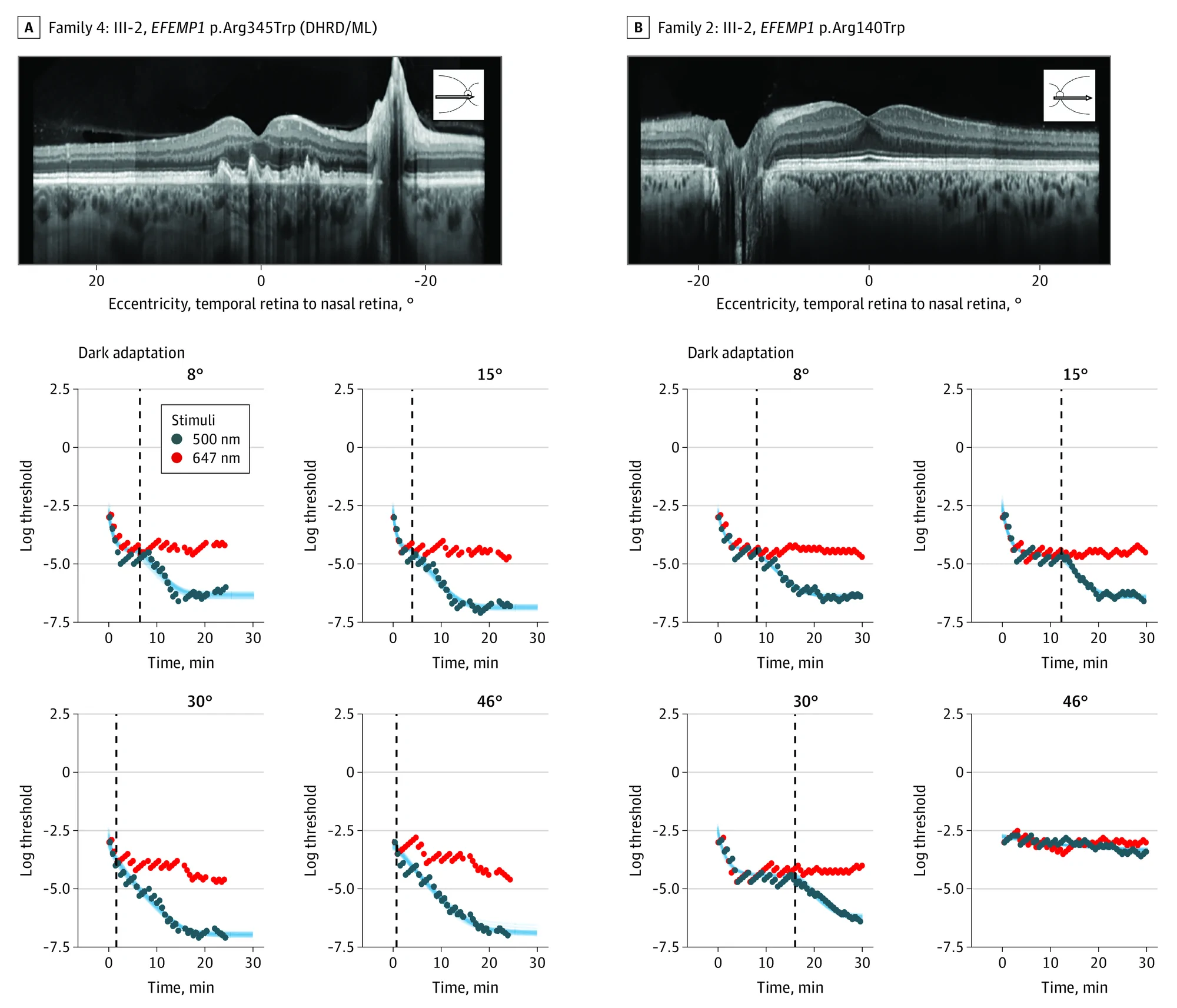
Functional Phenotype Assessed by Rod-Mediated Dark Adaptation Representative optical coherence tomography images (top) and dark adaptometry results (bottom) are shown for the same 2 patients as in Figure 1. Dark adaptation was measured at multiple retinal eccentricities (8°, 15°, 30°, and 46°) along the horizontal meridian in the temporal retina (corresponding to the nasal visual field), a region without clinically visible chorioretinal atrophy in both patients. The blue and red dots denote the blue (500 nm) and red (647 nm) stimuli seen by the patient. The dashed vertical line indicates the rod-cone break. Family 4, individual III-2 (Doyne honeycomb retinal dystrophy and malattia leventinese [DHRD/ML]) shows rod-mediated dark adaptation within normal limits at all tested eccentricities, consistent with preserved rod function outside the central macula. Family 2, individual III-2 (*EFEMP1* p.Arg140Trp) demonstrates severely delayed rod-mediated dark adaptation at 15°, 30°, and 46° eccentricities, with increasing severity toward the peripheral retina. Despite the absence of overt chorioretinal atrophy at these temporal retinal locations, rod recovery kinetics are markedly abnormal, indicating early and progressive rod dysfunction preceding structural degeneration.

To contrast disease expression of *EFEMP1* p.Arg140Trp retinal degeneration vs *EFEMP1* p.Arg345Trp DHRD/ML, retinal structure and function were examined using color fundus photography and SD-OCT imaging (Figure 1), steady-state light- and dark-adapted perimetry (Figure 2; photoreceptor-specific PERIRD subtests in eFigure 4 in Supplement 1), and dark adaptation kinetics following bright adapting lights (Figure 3). In family 4, patient III-2 (DHRD/ML) at age 53 years, fundus examination revealed the characteristic phenotype of central drusen-like deposits with associated hyperpigmentary changes, while SD-OCT demonstrated preservation of retinal lamination outside the central macula (Figure 1). Consistent with the limited spatial extent of structural involvement, light-adapted (cone-mediated) and dark-adapted (rod-mediated) static perimetry along the vertical meridian showed near-normal sensitivities outside the central 10° (diameter), indicating relatively preserved peripheral cone and rod function despite prominent macular abnormalities (Figure 2; eFigure 4 in Supplement 1).

In contrast, family 2, patient III-2, carrying the *EFEMP1* p.Arg140Trp variant, showed a distinct structure-function relationship. Fundus imaging demonstrated lobular (cobblestone-like) chorioretinal atrophic lesions in a semicircumferential distribution in the nasal retina at approximately 30° to 45° eccentricity from the fovea. In addition, a subtle yellowish outer retinal and subretinal deposit was visible in a near-circumferential band beginning at approximately 25° to 30° eccentricity and extending toward the far periphery. Corresponding SD-OCT, particularly along the inferior meridian, showed ellipsoid zone abnormalities from approximately 20° eccentricity extending peripherally (Figure 1).

Vertical meridian perimetry showed marked rod-mediated sensitivity loss peaking at approximately 20° in the superior visual field (inferior retina), in close proximity to the border of chorioretinal atrophy. S-cone sensitivity was also reduced at nearby loci, whereas L/M-cone sensitivity appeared relatively preserved (Figure 2; eFigure 4 in Supplement 1). Because these test locations lie near an atrophic transition zone, it remains uncertain whether the apparent L/M-cone sparing reflects true differential photoreceptor vulnerability or small torsional differences in eye position or head tilt during testing.

To characterize dynamic rod recovery, dark adaptometry was performed at multiple eccentricities (8°, 15°, 30°, and 46°) along the horizontal meridian in the temporal retina (nasal visual field), a region without clinically visible chorioretinal atrophy in patient III-2 of family 4 and patient III-2 of family 2 (Figure 3). The patient with DHRD/ML (family 4, individual III-2) showed rod-mediated dark adaptation within normal limits overall (and with a borderline delay at 8°), consistent with preserved peripheral rod function outside the macula. In contrast, the patient with *EFEMP1* p.Arg140Trp retinal degeneration (family 2, individual III-2) demonstrated severely delayed rod-mediated dark adaptation at all tested loci, with increasing severity toward the peripheral retina (delays in rod intercept time compared with normal data of 4.6 minutes at 8°, 7.5 minutes at 15°, and 10.2 minutes at 30°, with no rod recovery measurable within 60 minutes at 46°) (eFigure 5 in Supplement 1). These abnormalities were present in retinal regions that appeared structurally intact by fundus examination and OCT, indicating that rod dysfunction precedes or exceeds detectable structural degeneration.

In family 1, individuals III-1, III-3, and III-5, OCT cross-sections showed preservation of the outer nuclear layer along the horizontal and vertical meridians to ±30° eccentricity (eFigure 6 in Supplement 1). There was no evidence of macular drusen-like deposits. Despite this structural preservation, rod intercept time was delayed by 4.0 to 10.5 minutes at 30° eccentricity in the nasal retina (eFigure 5 in Supplement 1).

At 69 years, family 3, patient II-3 showed diffuse chorioretinal atrophy involving nearly the entire posterior pole with preservation of a central macular island (eFigure 3 in Supplement 1). Fundus autofluorescence demonstrated markedly reduced signal across the atrophic region with central sparing. OCT confirmed extensive outer retinal atrophy with a residual ellipsoid zone island measuring approximately 8° horizontally and 7° vertically, slightly greater superiorly than inferiorly. This phenotype closely resembles that of family 1, patient II-1 at age 68 years (see Milam et al^5^).

### Variant EFEMP1, Abnormal Intracellular Behavior, and Increased Multimerization In Vitro

**Figure 4.**
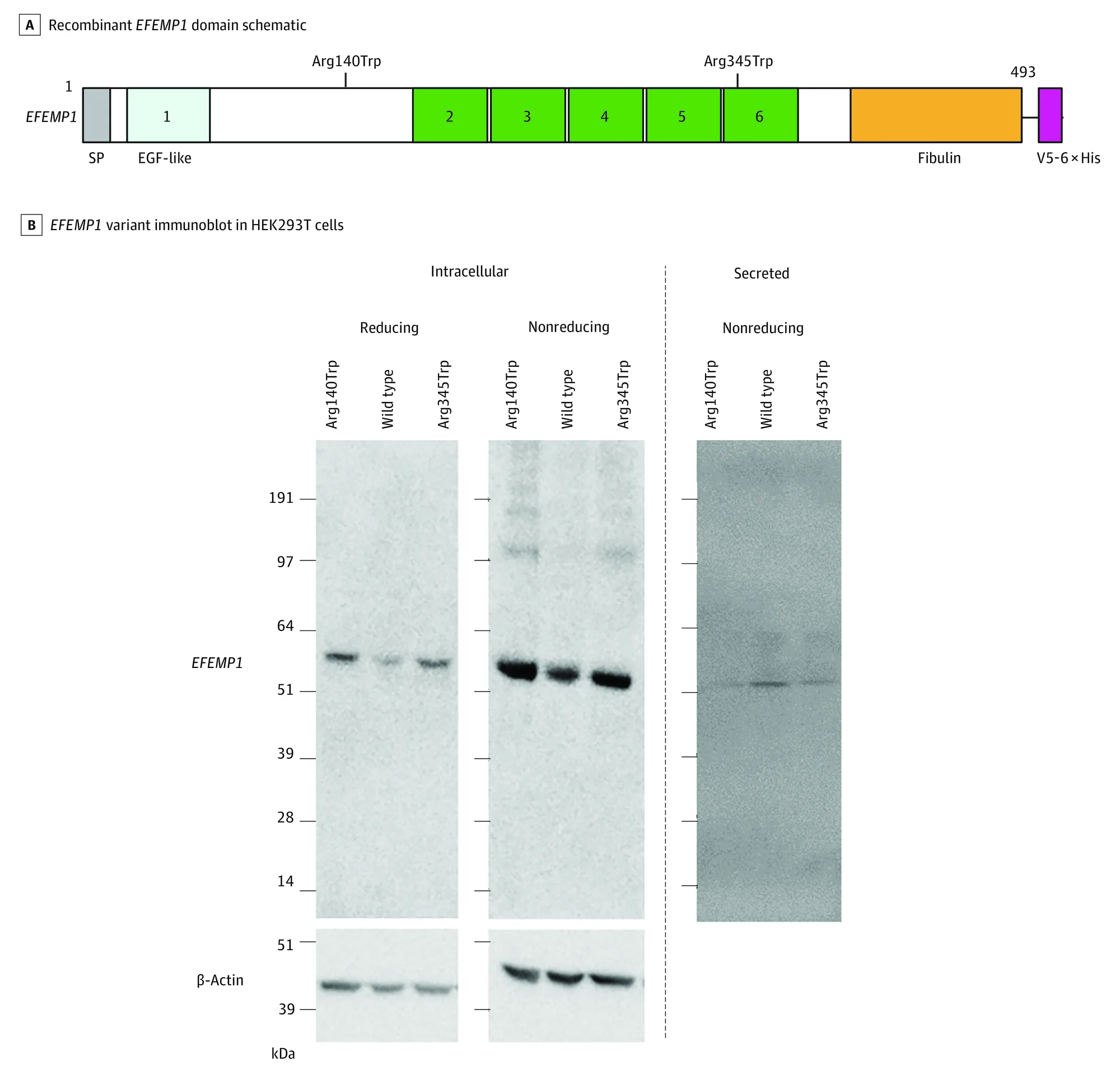
Domain Architecture and Immunoblot Analysis of *EFEMP1* Variants A, Schematic of recombinant *EFEMP1* (493 aa) with C-terminal V5-6 × His tag, showing domain organization for Uniprot entry Q12805 (gray indicates signal peptide [SP]; light blue indicates an atypical EGF domain; and green represents conventional Ca^2+^-binding epidermal growth factor [EGF] domains), and the positions of pathogenic variants p.Arg140Trp and p.Arg345Trp. B, Representative immunoblot of wild-type, p.Arg140Trp, and p.Arg345Trp *EFEMP1* after transient expression in HEK293T cells (which do not express endogenous *EFEMP1*). Under reducing conditions, both variant proteins show increased intracellular abundance relative to wild-type, consistent with impaired protein handling and turnover. Under nonreducing conditions, p.Arg140Trp demonstrates increased dimer (approximately 110 kDa) and higher-order oligomer (≥200 kDa) formation compared with wild-type. β-actin was probed as a loading control. Representative images from 3 independent experiments are shown. Densitometric quantification of these data are provided in Figure 5.

**Figure 5.**
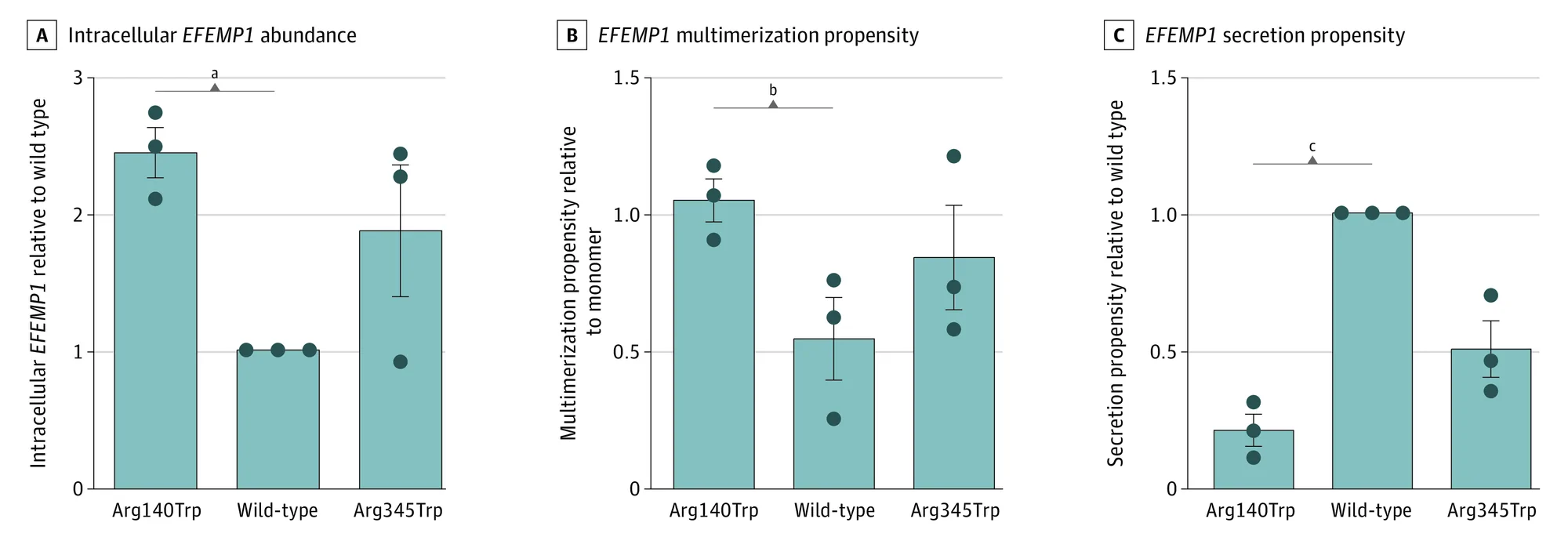
Bar Graphs Quantifying *EFEMP1* Intracellular Abundance, Multimerization, and Secretion Densitometric quantification of the immunoblot data shown in Figure 4B; bars indicate mean (SEM) from 3 independent experiments, while dots indicate experiments. A, Intracellular abundance of wild-type, p.Arg140Trp, and p.Arg345Trp *EFEMP1* under reducing conditions, normalized to β-actin. Both variant proteins show significantly increased intracellular abundance relative to wild-type, consistent with impaired protein handling and turnover. B, Multimerization propensity under nonreducing conditions, calculated as the ratio of intracellular dimer plus higher-order oligomer to monomer signal. p.Arg140Trp shows significantly increased multimerization compared with wild-type. C, Secretion propensity, calculated as the ratio of secreted to intracellular monomer signal under nonreducing conditions. Both p.Arg140Trp and p.Arg345Trp show a significant secretion defect compared with wild-type EFEMP1. ^a^*P* < .05, calculated using the Welch *t* test against a hypothetical mean of 1 (ie, unchanged) for wild-type (*t* [*df*], 7.85 [2]). ^b^*P* < .05, calculated using an unpaired 2-tailed *t* test (*t* [*df*], 2.97 [4]). ^c^*P* < .01, calculated using the Welch *t* test against a hypothetical mean of 1 for wild-type (t [df], 13.54 [2]).

The *EFEMP1* gene encodes the 493 amino acid fibulin-like extracellular matrix protein 1, also known as fibulin-3 (FBLN3 or EFEMP1) protein. Although function remains unclear, EFEMP1 is a secreted glycoprotein with important roles in the extracellular matrix. The p.Arg140Trp variant affects a conserved residue located in a linker region in the first, atypical calcium-binding epidermal growth factor (EGF)–like domain^13^ of EFEMP1 (Figure 4A; domains annotated according to Uniprot entry Q12805) and is predicted to be deleterious by multiple tools. To test its functional consequences in vitro, we transiently expressed human wild-type, p.Arg140Trp, or p.Arg345Trp EFEMP1 in HEK293T cells, which do not express endogenous EFEMP1. Compared with wild-type EFEMP1, both p.Arg140Trp (3 independent transfection experiments; Welch corrected *t* [*df*], 7.85 [2];* P* = .02) and p.Arg345Trp showed increased intracellular abundance on immunoblot under reducing conditions, consistent with abnormal protein handling (Figure 4B and Figure 5A). Under nonreducing conditions, p.Arg140Trp showed increased formation of intracellular dimers (approximately 110 kDa) and higher-order oligomers (≥200 kDa) compared with wild-type EFEMP1. Mean (SD) multimerization propensity for p.Arg140Trp, calculated by quantification of intracellular dimer and oligomer to monomer ratio, was 1.064 (0.138) compared with 0.553 (0.265) for wild-type EFEMP1 (unpaired *t* [*df*], 2.97 [4]; *P* = .04) (Figure 5B). Intracellular multimerization propensity of EFEMP1 p.Arg345Trp relative to monomer abundance was not significantly different from wild-type.

Misfolding, intracellular accumulation, and impaired secretion of p.Arg345Trp are well established; in our experiments, p.Arg140Trp also resulted in significantly impaired secretion into conditioned media collected 72 hours after transfection compared with wild-type EFEMP1 (3 independent transfection experiments; p.Arg140Trp 21, ±10%; Welch corrected *t* [*df*], 13.54 [2]; *P* = .005) (Figure 5C). This pattern is consistent with altered folding and aberrant intramolecular or intermolecular bonding arising from the p.Arg140Trp variant.

Alterations in native intramolecular disulfide bonding involving proximal cysteine residues in the sixth EGF domain significantly impact p.Arg345Trp monomer folding and impair secretion.^14^ Within the atypical EGF domain 1 of EFEMP1, there are 6 free cysteine residues (Cys48, 55, 61, 70, 159 and 171), each located some distance from residue 140 in EFEMP1 primary and predicted secondary structures (AlphaFold identifier AF-Q12805-F1), suggesting that steric hindrance or local changes in backbone conformation arising from the substitution of a large aromatic residue at 140 may not affect disulfide bond formation in the same way as p.Arg345Trp.

To further assess the mechanism of aberrant p.Arg140Trp dimerization, we performed in silico mutagenesis and interface modeling of EFEMP1 using AlphaFold 3 and Rosetta software. A plausible dimer configuration placed residue 140 at a subunit interface adjacent to aromatic residues (Tyr419 and Phe476) on the opposing chain; replacement of arginine by tryptophan was predicted to stabilize this interface through favorable aromatic packing interactions in both homomeric variant (free energy range, −2.71 to −3.79 kcal/mol) and heteromeric variant and wild-type complexes (free energy range, −1.35 to −1.89 kcal/mol) (eFigure 7 in Supplement 1).^15^ Together with the biochemical findings of intracellular accumulation and impaired secretion, these computational results suggest that p.Arg140Trp perturbs EFEMP1 quaternary assembly and increases propensity for abnormal intracellular multimerization.

## Discussion

This case series identified *EFEMP1* p.Arg140Trp as the cause of a distinct late-onset retinal degeneration. Across 3 unrelated families, the disease was characterized by severe rod dysfunction, often detectable decades before overt structural degeneration, relative preservation of central vision, and a peripheral-predominant spatial pattern of functional loss. These features distinguish it from both canonical *C1QTNF5*-associated L-ORD and *EFEMP1*-associated DHRD/ML.

The topography of disease in *EFEMP1* p.Arg140Trp retinal degeneration is strikingly different from that of classic DHRD/ML. Whereas *EFEMP1* p.Arg345Trp produces a macular- and peripapillary-centered deposit phenotype with relative peripheral sparing,^16,17^ *EFEMP1* p.Arg140Trp shows an inverse pattern with peripheral-predominant dysfunction and atrophy and relative macular preservation. The combination of late-onset disease together with macular preservation caused by the p.Arg140Trp variant may result in less awareness of visual symptoms compared with other adult-onset diseases with macular involvement such as L-ORD, DHRD/ML, and Sorsby fundus dystrophy. Also in line with distinct mechanisms, no CNV was detected clinically in any p.Arg140Trp carrier across families 1 to 3, in contrast with DHRD/ML, in which a CNV prevalence of approximately 27% has been reported.^18^ However, the small sample size in our cohort limits inferences about the true CNV frequency in *EFEMP1* p.Arg140Trp–associated retinal degeneration.

Contrasting topographies between 2 EFEMP1 variants imply variant-specific effects on the protein, altering sub-RPE extracellular environment and deposit formation. The basis for regional susceptibility remains unresolved but the divergence is compatible with cell-culture observations. Both EFEMP1 p.Arg345Trp and p.Arg140Trp variant proteins showed intracellular accumulation and impaired secretion in our study. Altered disulfide bonding in the sixth EGF domain renders EFEMP1 p.Arg345Trp intrinsically unstable, resulting in misfolding and intracellular retention within the endoplasmic reticulum, leading to activation of the unfolded protein response pathway and increasing RPE cell stress.^14,19^ A secretion defect is apparent; however, a proportion of EFEMP1 p.Arg345Trp is secreted by cells in in vitro studies, including by primary RPE derived from *Efemp1* R345W knock-in mice and patient-derived induced pluripotent stem cells differentiated to RPE, and contributes to deposit formation.^20,21^

EFEMP1 p.Arg140Trp is less well characterized and has been reported to be indistinguishable from wild-type in secretion and accumulation assays^22^ or, as we and others observed, to accumulate intracellularly and form intracellular dimers.^23,24^ We extend reports that EFEMP1 dimers are detectable under native conditions but are difficult to preserve after sodium dodecyl sulfate-polyacrylamide gel electrophoresis,^25,26^ and support the possibility that p.Arg140Trp alters folding or cysteine accessibility at the EFEMP1 N-terminus to promote aberrant intermolecular interactions. In silico modeling supports this interpretation by identifying a plausible interface in which Trp140 can significantly stabilize dimer packing near Tyr419/Phe476 compared with wild-type EFEMP1, thus impairing secretion (eDiscussion in Supplement 1).

Notably, p.Arg140Trp has also been associated with primary open-angle glaucoma without reported retinal abnormalities, underscoring uncertainty regarding tissue specificity, penetrance, or modifying factors.^23^ In the present study, 2 of 3 families showed a history of ocular hypertension or glaucoma in some, but not all carriers (eTable in Supplement 1).

Clinically, a central finding is the marked dissociation between retinal structure and function. Abnormal recovery kinetics and rod dysfunction occurred at retinal locations with a preserved outer nuclear layer and RPE, indicating dysfunction that precedes detectable photoreceptor loss. Given this therapeutic window and recent evidence that complement factor B inhibition reduces sub-RPE deposit formation in an Efemp1 knock-in mouse model, treatment trials warrant consideration.^27^ Moreover, because p.Arg140Trp—like the canonical p.Arg345Trp variant—is a single recurrent missense substitution, allele-specific approaches, including antisense oligonucleotides, may offer alternative therapeutic avenues; analogous strategies have already been explored for the related p.Arg345Trp allele.^21^ Our findings raise the possibility that *EFEMP1* variants contribute to other L-ORD–like phenotypes, including diffuse-trickling geographic atrophy^28^ and extensive macular atrophy with pseudodrusen-like appearance,^29^ in which previous screening for *C1QTNF5* variants did not yield a genetic diagnosis.^30,31^

### Limitations

This study has several limitations. Clinical and genetic characterizations derive from only 3 unrelated families, so the phenotypic spectrum and the apparent absence of choroidal neovascularization should be interpreted with caution and may not capture the full range of disease expression. In addition, the functional data rely on an overexpression system that does not fully recapitulate the native RPE environment, and the in-silico interface modeling awaits experimental validation. Thus, the proposed p.Arg140Trp dimerization mechanism remains provisional.

## Conclusions

In summary, *EFEMP1* p.Arg140Trp-associated retinal degeneration defines a peripheral-predominant, rod-vulnerable retinal degeneration distinct from both L-ORD and DHRD/ML. Topographically matched structural and functional assessments indicate a structure-function dissociation, implying that early treatment may prevent irreversible degeneration.

## Notes

Supplement 1. eTable. Patient Demographics, Genetics, and Ocular Findings eMethods. eFigure 1. Pedigree of Affected Families eFigure 2. Genetic Testing eFigure 3. Retinal Imaging in Family 3: II-3 eFigure 4. Photoreceptor-Specific Visual Field Testing in Malattia Leventinese Compared to *EFEMP1*:c.418C>T Retinal Degeneration eFigure 5. Delays in Rod-Mediated Dark Adaptation eFigure 6. Optical Coherence Tomography Cross-Sections of Family 1 Generation III eFigure 7. In Silico Dimer-Interface Modeling of *EFEMP1* p.Arg140Trp eDiscussion. eReferences.

Supplement 2. Data Sharing Statement
