# Supplementary material for: Widening the Spectrum of Disease Expression due to Heterozygous Variants in EFEMP1
Source: JAMA Ophthalmol. 2026 Sep 10;144(10):915–24. doi: 10.1001/jamaophthalmol.2026.3828 (PMC13563294; doi:10.1001/jamaophthalmol.2026.3828)
Supplement: Supplementary file 1 [file jamaophthalmol-e263828-s001.pdf]

Supplemental Online Content

Stanton CM, Ansari G, Pfau K, et al. Widening the spectrum of disease expression due to heterozygous variants in *EFEMP1*. *JAMA Ophthalmol.* Published online September 10, 2026. doi:10.1001/jamaophthalmol.2026.3828

eTable. Patient Demographics, Genetics, and Ocular Findings

eMethods.

eFigure 1. Pedigrees of the Affected Families

eFigure 2. Genetic Testing

eFigure 3. Retinal Imaging in Family 3: II-3

eFigure 4. Photoreceptor-Specific Visual Field Testing in Malattia Leventinese Compared to *EFEMP1*:c.418C>T Retinal Degeneration

eFigure 5. Delays in Rod-Mediated Dark Adaptation

eFigure 6. Optical Coherence Tomography Cross-Sections of Family 1 Generation III

eFigure 7. In Silico Dimer-Interface Modeling of *EFEMP1* p.Arg140Trp

eDiscussion.

eReferences.

This supplemental material has been provided by the authors to give readers additional information about their work.

© 2026 Stanton CM et al. *JAMA Ophthalmology*.

eTable. Patient demographics, genetics, and ocular findings.

Family	Generation-Subject ID	Sex	Age at Exam (yrs)	Age of Symptom Onset	Zygosity	Nucleotide Change (Reference Sequence: NM_001039348.3)	BCVA	ERG	Dark Adaptation	Other Key Findings / Notes	Evidence of Glaucoma and/or Ocular Hypertension
F1	I-1	M	—	—	—	Not known	Not known	Not known	Not known	No reported visual symptoms; died at age 78	Not known
F1	I-2	F	—	—	—	Not known	Not known	Not known	Not known	No reported visual symptoms; died at age 70	Not known
F1	II-1	M	68 (clinical); 82 (death)	50s	—	Not known	20/25 OU (age 68)	Abnormal	Not recorded	Annular midperipheral scotoma; described as similar to gyrate atrophy; postmortem histopathology	IOPs 27 at age 65; treated with propine
F1	II-2	F	70	Not known	—	Not known	Not recorded	Not recorded	Not recorded	Fundus described as similar to II-1; died at age 73	Not known
F1	III-1	M	50, 57	—	hetero	c.418C>T p.Arg140Trp	20/20 OU	Normal	Abnormal	Delayed rod adaptation; rare yellow dots, otherwise normal exam	IOPs 12/12
F1	III-2	F	53*	—	homo	WT	20/20 OU	Normal	Normal	Only generation-III subject without dark adaptation delay	IOP reported as normal
F1	III-3	M	51	—	hetero	c.418C>T p.Arg140Trp	20/20 OU	Normal	Abnormal	Delayed rod adaptation; otherwise normal fundus	Not recorded
F1	III-4	F	58	—	hetero	c.418C>T p.Arg140Trp	20/20 OU	Normal	Abnormal	Delayed rod adaptation	IOP reported as normal
F1	III-5	F	44, 50	45	hetero	c.418C>T p.Arg140Trp	20/20 OU	Normal	Abnormal	Mild drusen-like lesions; peripheral pigment changes	Glaucoma workup with IOPs of 19/20 at age 39; glaucoma suspect with IOPs of 20/20 at age 44, 19/20 at age 50

© 2026 Stanton CM et al. *JAMA Ophthalmology.*

F2	III-2	F	42	40	hetero	c.418C>T p.Arg140Trp	20/20 OU	Abnormal	Abnormal	Initially diagnosed as 'gyrate atrophy- or choroideremia-like' IRD with lobular atrophic lesions in the peripheral retina	No, intraocular pressure within normal limits
F3	II-3	F	69	55	hetero	c.418C>T p.Arg140Trp	20/32 OU	—	—	Noted nyctalopia when aged 55 yrs. Phenotype resembled severe choroideremia. Metabolic screening for gyrate atrophy was performed; plasma testing showed borderline alanine levels.	Treated for open-angle glaucoma since age 53 years with topical timolol (Arutimol) and latanoprost. Bilateral cataract surgery at age 66 years.
F4	III-2	M	53	53 (incidental finding)	hetero	c.1033C>T p.Arg345Trp	20/20 OD, 20/35 OS	—	Normal (except for a borderline delay at 8° eccentricity)	Doyne honeycomb retinal dystrophy/Malattia Leventinese (DHRD/ML) phenotype	No, intraocular pressure within normal limits

Abbreviations: heterozygous (hetero), homozygous (homo), best-corrected visual acuity (BCVA), electroretinography (ERG), intraocular pressure (IOP), inherited retinal disease (IRD)

** Available records indicate an age of 53 years at exam (previously listed as 46).*

© 2026 Stanton CM et al. *JAMA Ophthalmology*.

eMethods

Genetic testing

For family 1, whole-genome sequencing (WGS) of 2 affected and one unaffected generation III individuals was performed by Edinburgh Genomics (see below for segregation analysis among all generation III individuals). Libraries were prepared using the Illumina TruSeq PCR-free protocol and sequenced using an Illumina HiSeq X platform. BCBio-Nextgen (0.9.7) was used to perform alignment to Homo sapiens genome hg38, BAM file preparation and variant detection. Genotype likelihoods were calculated and VCF files created using Genome Analysis Toolkit (3.4-0-7e26328) Haplotype Caller. The individual VCFs were then screened using the G2P plugin,[2] and a version of the Eye Disorders panel (Nov, 2019)[3] to identify variants satisfying the gene inheritance model and variant frequency in general population.

Genotypes and segregation with disease in all generation III individuals were verified by PCR using primers (5'-3') F: AATTATCATCATCCTCACCACGA, R: GGATACCTCTGCCTTCCGAA, followed by Sanger sequencing and restriction digest using HpaII (NEB, R0171) of the 393 bp product, to generate fragments of 172, 100, 75, 40 bp in homozygous C (p.Arg140) samples, and 248, 172, 100, 75 and 45 bp for the C > T (p.Trp140) allele.

Bioinformatics

The Ensembl Variant Effect Predictor (Ensembl VEP[4]) was used to predict the deleteriousness of p.R140W on EFEMP1, using the integrated in silico tools SIFT, PolyPhen, ClinPred, REVEL, AlphaMissense, CADD, BLOSUM62 and EVE.

EFEMP1 cloning and plasmid preparation

Full-length *EFEMP1* was amplified by PCR from human iPSC-RPE cDNA using PrimeSTAR HS DNA polymerase (Takara) and gene-specific attB-flanked PCR primers (5'-3') F: GGGGACAAGTTTGTACAAAAAAGCAGGCTTCACC ATGTTGAAAGCCCTTTTCCT, R: GGGGACCACTTTGTACAAGAAAGCTGGGTCAAATGAAAATGGCCCCACTA. After gel purification (Qiagen), the attB-flanked *EFEMP1* was used in a Gateway BP recombination reaction with the donor vector pDONR221, and then moved into pcDNA-DEST40 via Gateway LR recombination as described by the manufacturer (ThermoFisher Scientific). Plasmids were propagated in TOP10 E. coli (Invitrogen) and purified using a plasmid miniprep kit (Qiagen). EFEMP1 p.Arg140Trp and p.Arg345Trp variants were made in the entry vector using the QuikChange Site Directed mutagenesis II kit (Stratagene), according to the manufacturer's protocol and using the following primers: Arg140Trp (c.418C>T, 5'-gaaataactttgtcatctggcggaacccagctga-3', 5'-tcagctgggttccgccagatgacaaagttatttc-3'; Arg345Trp (c.1033C>T), 5'-gagaccacaaatgaatgcagggaggatgaaatgtgtt-3', 5'-aacacatttcatcctccctgcattcatttgtggtctc-3'. EFEMP1 insert and mutations were verified by Sanger sequencing. Plasmids for mammalian cell transfections were purified using HiPure plasmid kits (Invitrogen).

Cell culture and transfection

HEK293T cells were maintained in Dulbecco's Modified Eagle Medium (DMEM) containing GlutaMAX (ThermoFisher Scientific) supplemented with 10% (v/v) fetal bovine serum (Gibco) and 1% penicillin–streptomycin (Gibco) at 37 °C in a humidified atmosphere containing 5% CO_2. HEK293T cells tested negative for mycoplasma. For transfection, cells were seeded in 6-well plates and grown to ~70–80% confluence. For each reaction, 4 µg of plasmid DNA was transfected using 10 µL of Lipofectamine 2000 (Thermo Fisher Scientific) according to the manufacturer's instructions. Medium was replaced with serum-free medium after 6 h. 72 h post-transfection, conditioned media was collected, centrifuged at 1200 rpm for 10 minutes at 4 °C and aliquoted for storage at -70 °C until use. Cells were washed with PBS, and cell lysates were prepared in RIPA buffer (150 mM NaCl, 50 mM Tris pH 8, 1% NP-40, 0.5% sodium deoxycholate, 0.1% SDS) and stored at -70 °C until use. Transfections were performed a minimum of three times.

© 2026 Stanton CM et al. *JAMA Ophthalmology*.

Immunoblotting

Cell lysates were centrifuged at 10,000 x g, 10 min before soluble protein concentration was determined using Bio-Rad Protein Assay. Under non-reducing or reducing (1 × Reducing Agent, Invitrogen) conditions, 20 µg of soluble protein or 20 µl of conditioned media was mixed with 4 × NuPAGE LDS Sample Buffer. Proteins and SeeBlue Plus2 molecular weight marker (Invitrogen) were separated on 4-12% Bis-Tris NuPAGE gels (Invitrogen) run in 1 × MOPS (3-(N-morpholino)propanesulfonic acid; Invitrogen) then transferred onto PVDF as described previously.[5,6] Membranes were probed with rabbit anti-EFEMP1 (1:1000, HPA070841, Atlas Antibodies), followed by anti-rabbit IgG horseradish peroxidase (HRP)-conjugated secondary antibodies. Protein bands were visualized using an ImageQuant LAS 4000 (GE Healthcare) following addition of Enhanced Chemiluminescence (ECL) Western blotting detection reagents (Cytiva). Membranes were stripped using Restore Western Blot Stripping Buffer (ThermoFisher) for reprobing with mouse anti-β actin (1:5000, T5441, Sigma) detected by anti-mouse IgG HRP-conjugated secondary antibodies, as a loading control. Densitometric analysis was carried out using Image Studio Lite software (LI-COR). Statistical significance was evaluated using unpaired two tailed t-test with Welch's correction if standard deviation (SD) for each group were not equal (GraphPad Prism 10.6.1) based on data from three independent experiments.

Structural modelling and in silico biophysical variant effect evaluation

A local installation of AlphaFold 3 (AF3), a generative structure prediction tool, was used to derive possible EFEMP1 dimer structures.[7] To more closely simulate the native context, wild-type EFEMP1 sequences (Uniprot entry Q12805) were modelled with a NAG-NAG-BMA-(MAN)2 glycan attached at Asn249.[8–10] AF3 was run using seeds 1 through 15, prioritizing models where the sidechain of Arg140 occurred at the dimer interface. This approach identified a dimer structure with Arg140 colocalized in proximity to Tyr419/Phe476 of partner subunit. The model was used as a starting point for downstream biophysical evaluation.

The impact of p.Arg140Trp on dimer stability was calculated via the Rosetta cartddg protocol after removing glycans to ensure pipeline compatibility.[11–13] The structure was first energetically pre-minimized using the relax application (flags: -use_input_sc -ex1 -ex2 -nstruct 50 -no_optH false -flip_HNQ -score:weights ref2015_cart -relax:min_type lbfgs_armijo_nonmonotone -relax:script cart2 -fa_max_dis 9.0). The lowest energy structure was subsequently selected for mutagenesis with the cartesian_ddg application (flags: -ddg:iterations 3 -ddg::score_cutoff 1.0 -ddg::cartesian -ddg:bbnbrs 1 -fa_max_dis 9.0 -missing_density_to_jump -score:weights ref2015_cart -force_iterations false -ddg::legacy false). The p.Arg140Trp variant was introduced to both chains. The resulting lowest energy mutant and wild-type structures were relaxed again, producing 50 models each. The ΔΔG final was calculated as the difference between the average mutant and wild-type energies, with negative values indicating a stabilizing effect. The stability of the heterodimer (mutant/wild-type) was approximated as half of the calculated ΔΔG.

eFigure 1. Pedigrees of the Affected Families

In family 1, the hashed symbols denote individuals with delayed dark adaptation kinetics. However, there was no (clinical) evidence of outer retinal degeneration at the time of presentation.

Dashed symbols indicate uncertainty regarding disease status. In families 2 and 3, individuals that are potentially heterozygous were unavailable for clinical examination or asymptomatic genetic testing.

Family 1 (Philadelphia)

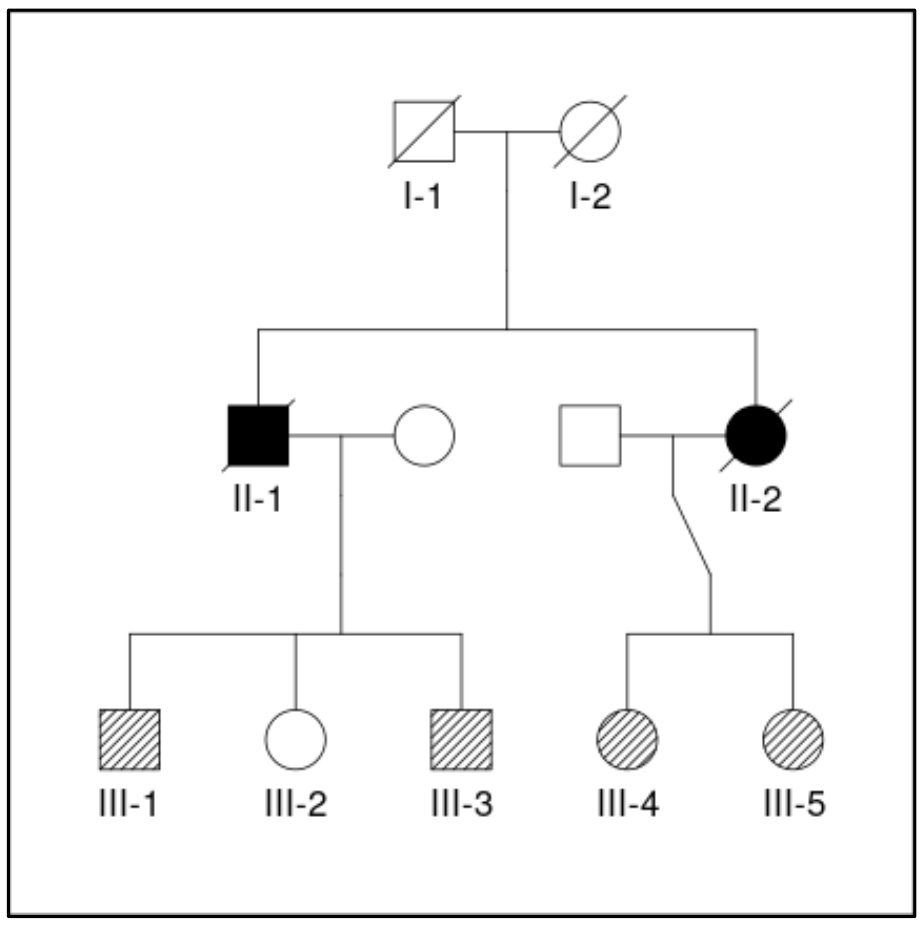

Family 2 (Basel)

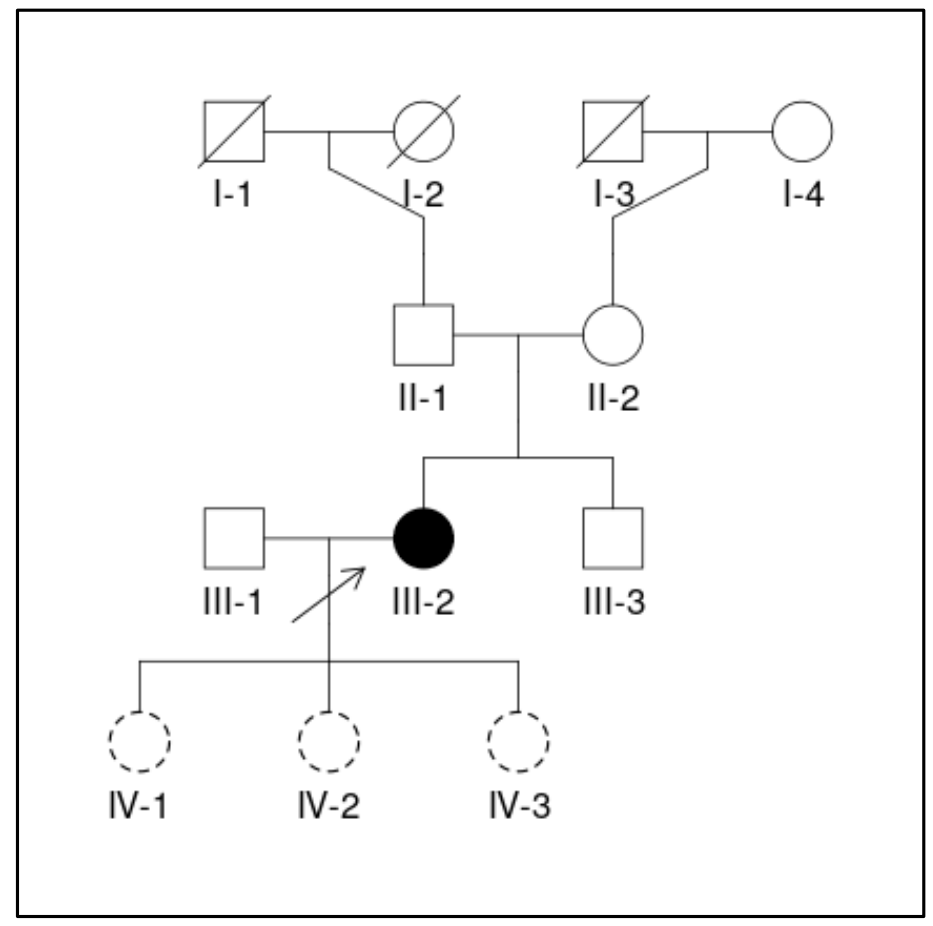

Family 3 (Prague)

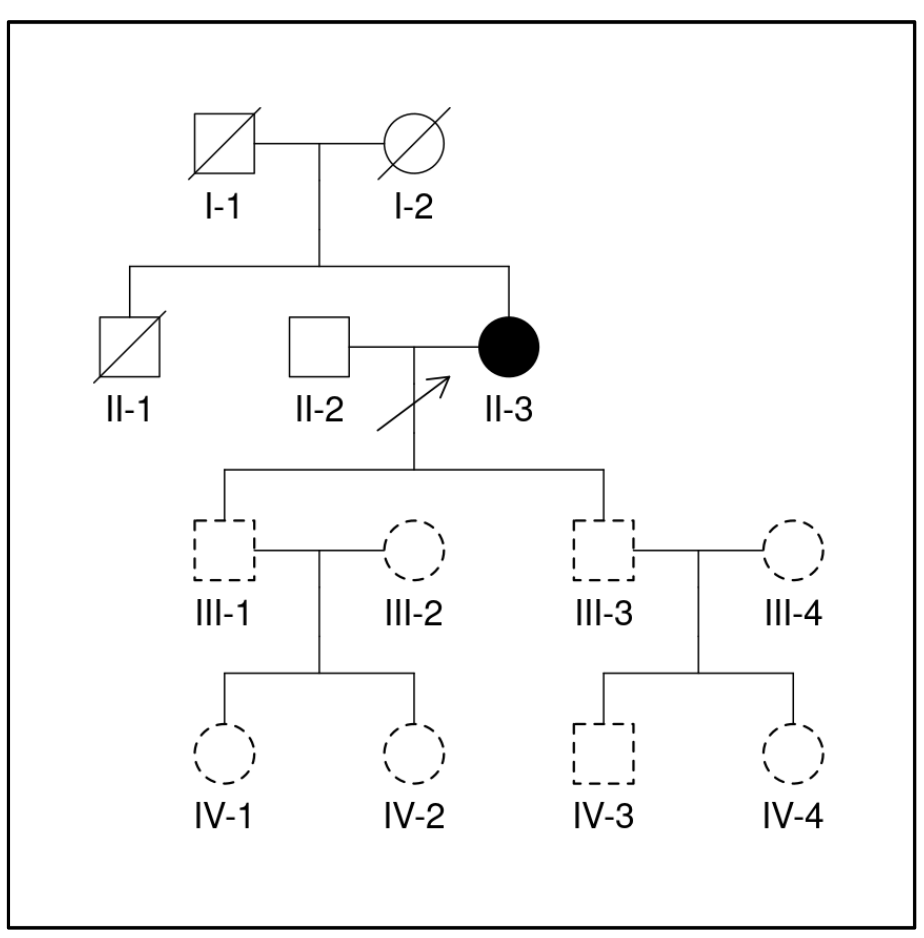

Family 4 (Basel) – DHRD/ML

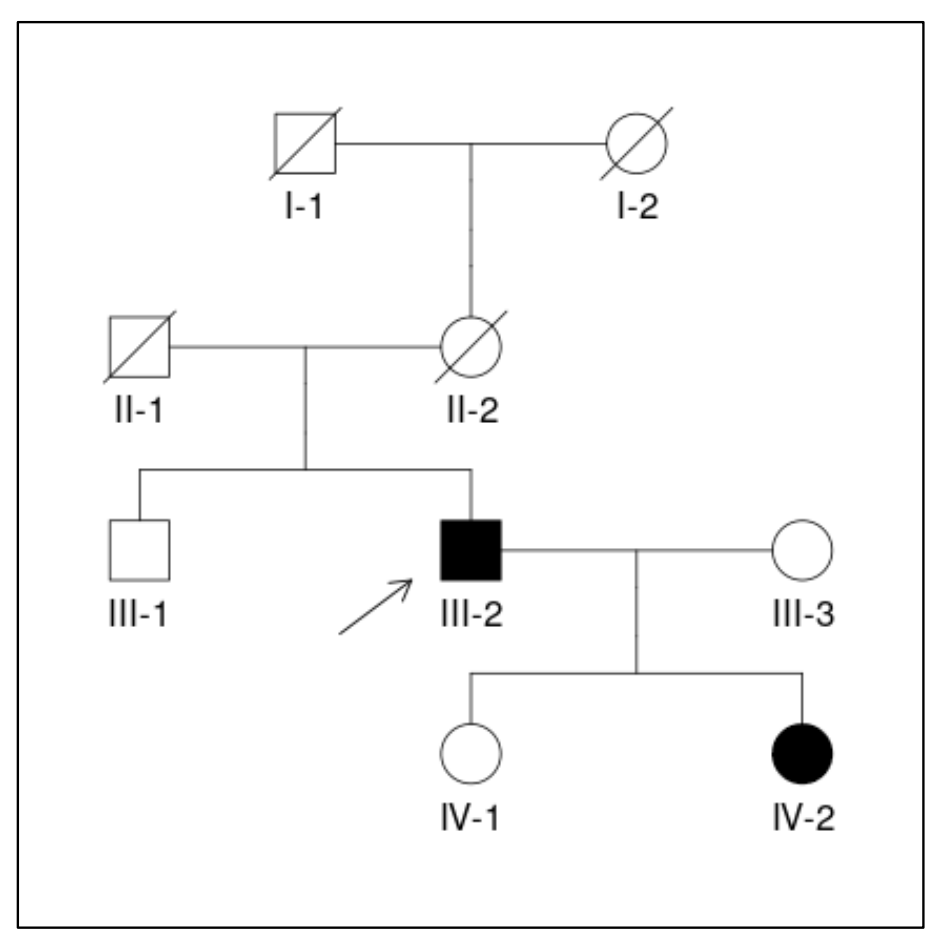

© 2026 Stanton CM et al. *JAMA Ophthalmology.*

eFigure 2. Genetic Testing

(**A**) Representative Sanger sequencing of *EFEMP1* exon 5 identifies the c.418C>T transition in affected members of Family 1, corresponding to p.Arg140Trp. (**B**) A representative image of HpaII restriction digest used to confirm heterozygosity for the variant in affected individuals.

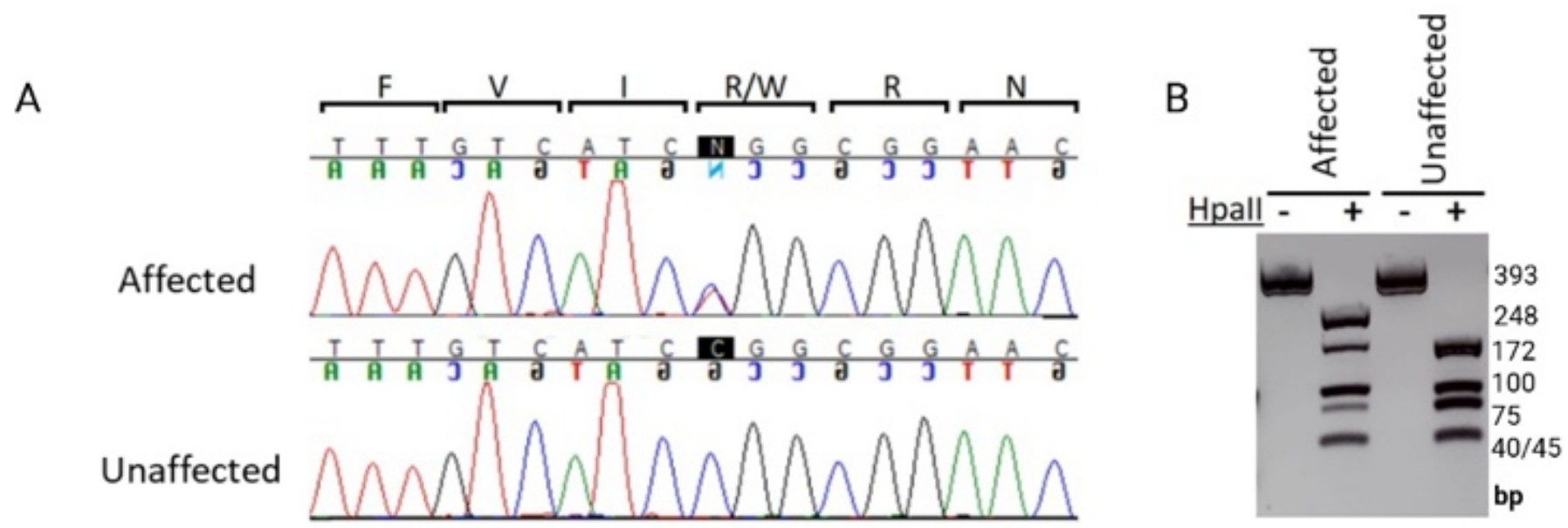

© 2026 Stanton CM et al. *JAMA Ophthalmology.*

eFigure 3. Retinal Imaging in Family 3: II-3

Color fundus photography (first row) shows diffuse atrophy involving nearly the posterior pole, with preservation of a central macular island. Short-wavelength fundus autofluorescence (second row) reveals markedly decreased signal throughout the posterior pole, consistent with absence of retinal pigment epithelium, except for a preserved central macular area. Optical coherence tomography (third and fourth row) cross-sections demonstrate extensive outer retinal atrophy, with a residual island of intact ellipsoid zone measuring approximately 8° horizontally and 7° vertically. Along the vertical meridian, the superior extent of the preserved ellipsoid zone is slightly greater than the inferior extent.

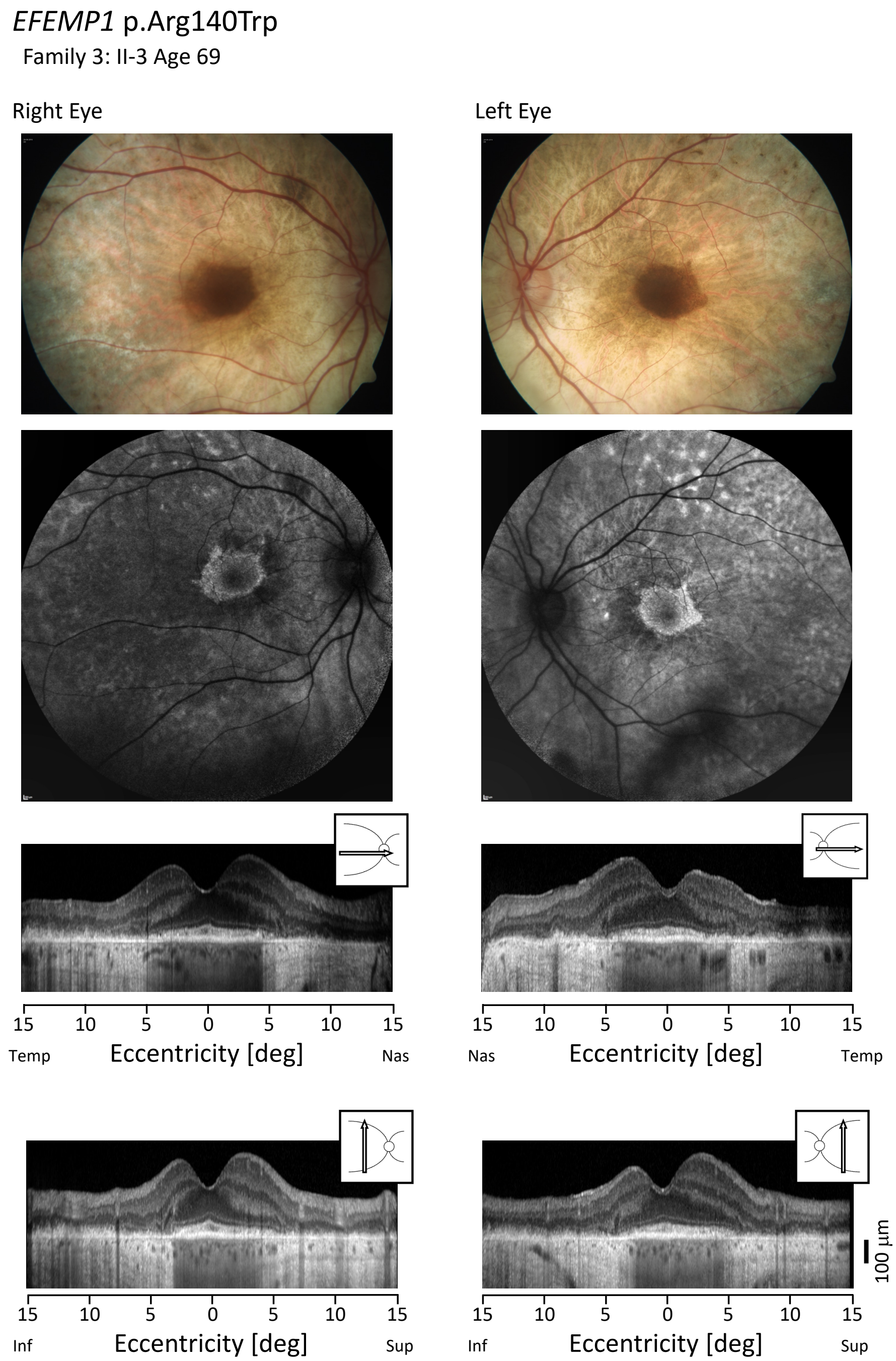

© 2026 Stanton CM et al. *JAMA Ophthalmology*.

eFigure 4. Photoreceptor-Specific Visual Field Testing in Malattia Leventinese Compared to *EFEMP1* p.Arg140Trp Retinal Degeneration

Static perimetry along the vertical meridian is shown for the same two patients as in Figure 1, using the full PERIRD (perimetry in retinal degeneration) protocol to isolate L/M-cone-, S-cone-, and rod-mediated function. Test conditions include light-adapted white-on-white, light-adapted blue-on-yellow (S-cone–mediated), red-on–dim blue (L/M-cone), and dark-adapted (rod-mediated) stimuli.

In **Family 4, individual III-2 (Doyne honeycomb retinal dystrophy/Malattia Leventinese [DHRD/ML])**, light-adapted (cone-mediated) and dark-adapted (rod-mediated) static perimetry along the vertical meridian indicate near-normal cone and rod sensitivity outside the central 10° (diameter), consistent with relatively preserved peripheral photoreceptor function despite prominent macular pathology.

Family 2, individual III-2 shows nominally greater rod-mediated sensitivity loss on dark-adapted testing, with a peak near 20° in the superior visual field (inferior retina). Light-adapted blue-on-yellow testing suggests co-localized S-cone involvement at nearby loci, while light-adapted white-on-white and red-on-dim blue measures appear comparatively less affected. However, small torsional differences in eye position or head tilt could contribute to the apparent rod–cone dissociation, because these locations are close to the atrophic border.

Abbreviations: DHRD/ML (Doyne honeycomb retinal dystrophy/Malattia Leventinese), R (rod-mediation of dark-adapted blue and red stimulus), M (mixed mediation of the dark-adapted blue [rod] and red [cone] stimulus)

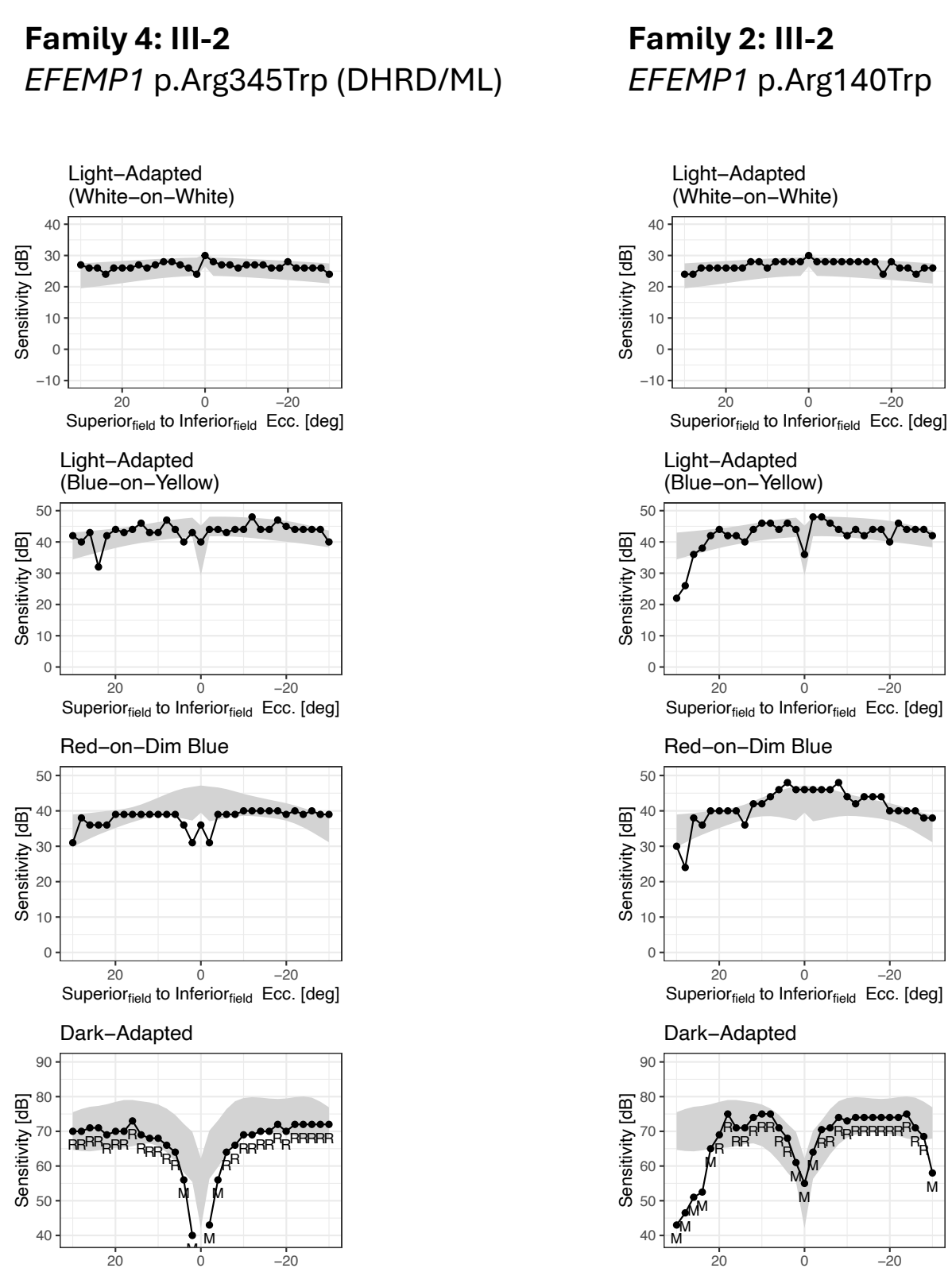

© 2026 Stanton CM et al. *JAMA Ophthalmology.*

eFigure 5. Delays in Rod-Mediated Dark Adaptation

The plot shows the delay in rod-mediated dark adaptation as a function of eccentricity. The plot includes data for Family 2: III-2 and previously published data from Family 1 Generation III. To account for differences in bleach intensity and in the definition of the criterion threshold to determine the rod-intercept time (RIT), all RIT values were converted to RIT delays (i.e., difference from the normative mean at each site). The error bars for F1 (III-1, -3, -4, and -5) denote the standard deviation. In addition, dark adaptation data from family 4, individual III-2 (Doyne honeycomb retinal dystrophy/Malattia Leventinese [DHRD/ML]) are shown as a comparator.

In Family 1, sensitivity recovery was assessed at retinal loci corresponding to 4° (nasal retina), 12° (inferior retina), and 30° (nasal retina). In Family 2: III-2, dark adaptometry was assessed at retinal eccentricities of 8°, 15°, 30°, and 46°, all located in the temporal retina.

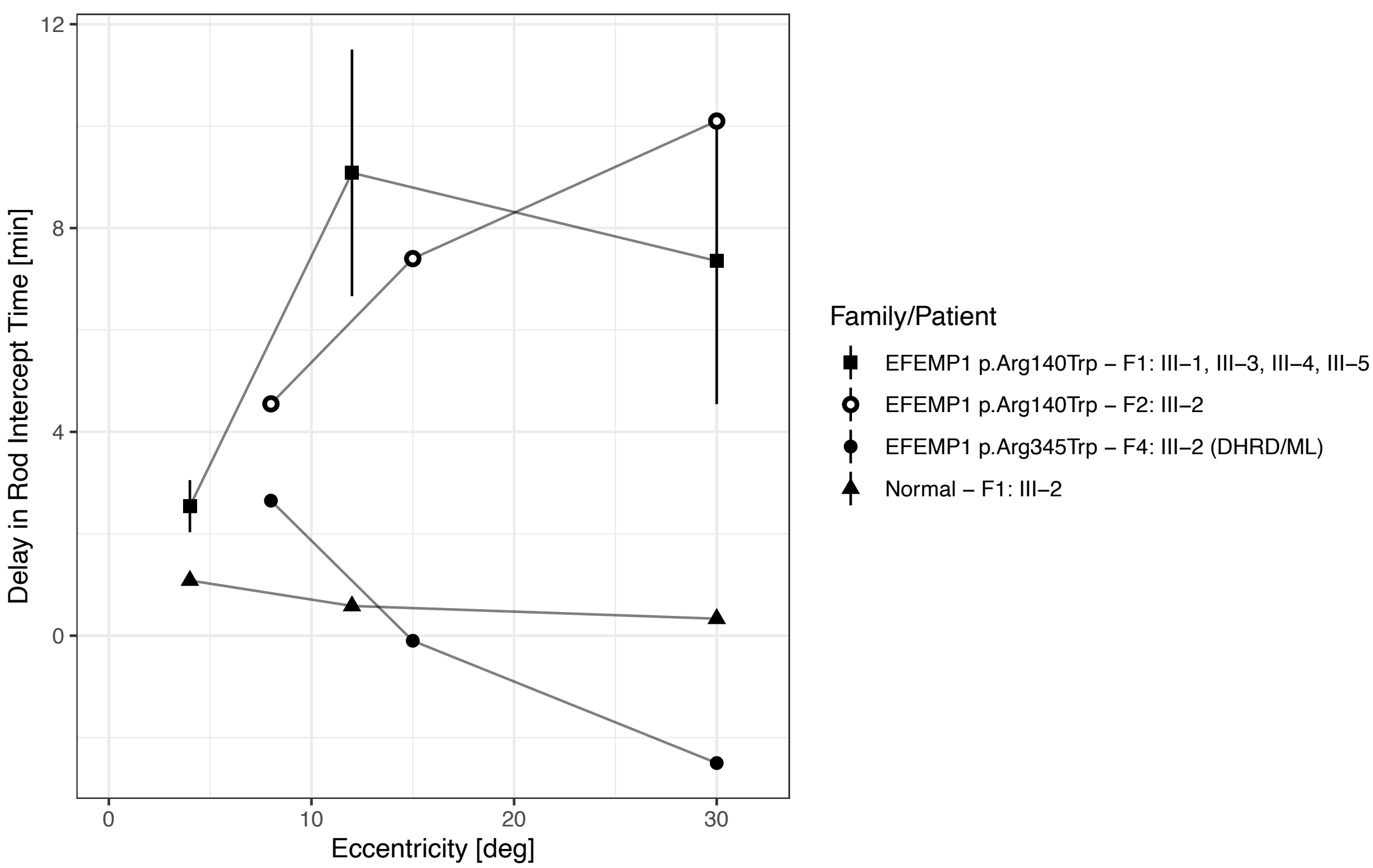

© 2026 Stanton CM et al. *JAMA Ophthalmology.*

eFigure 6. Optical Coherence Tomography Cross-Sections of Family 1 Generation III

Despite dark adaptation delays, no abnormalities in retinal structure (including no macular drusen at ages 50-57 years) were evident within the central 60° for Family 1 members III-1, III-3, and III-5 carrying the *EFEMP1* p.Arg140Trp variant. Importantly, the affected individuals show no macular drusen-like deposits.

Abbreviations: ON (Optic Nerve), Temp (temporal), Nas (nasal), Sup (superior), Inf (inferior)

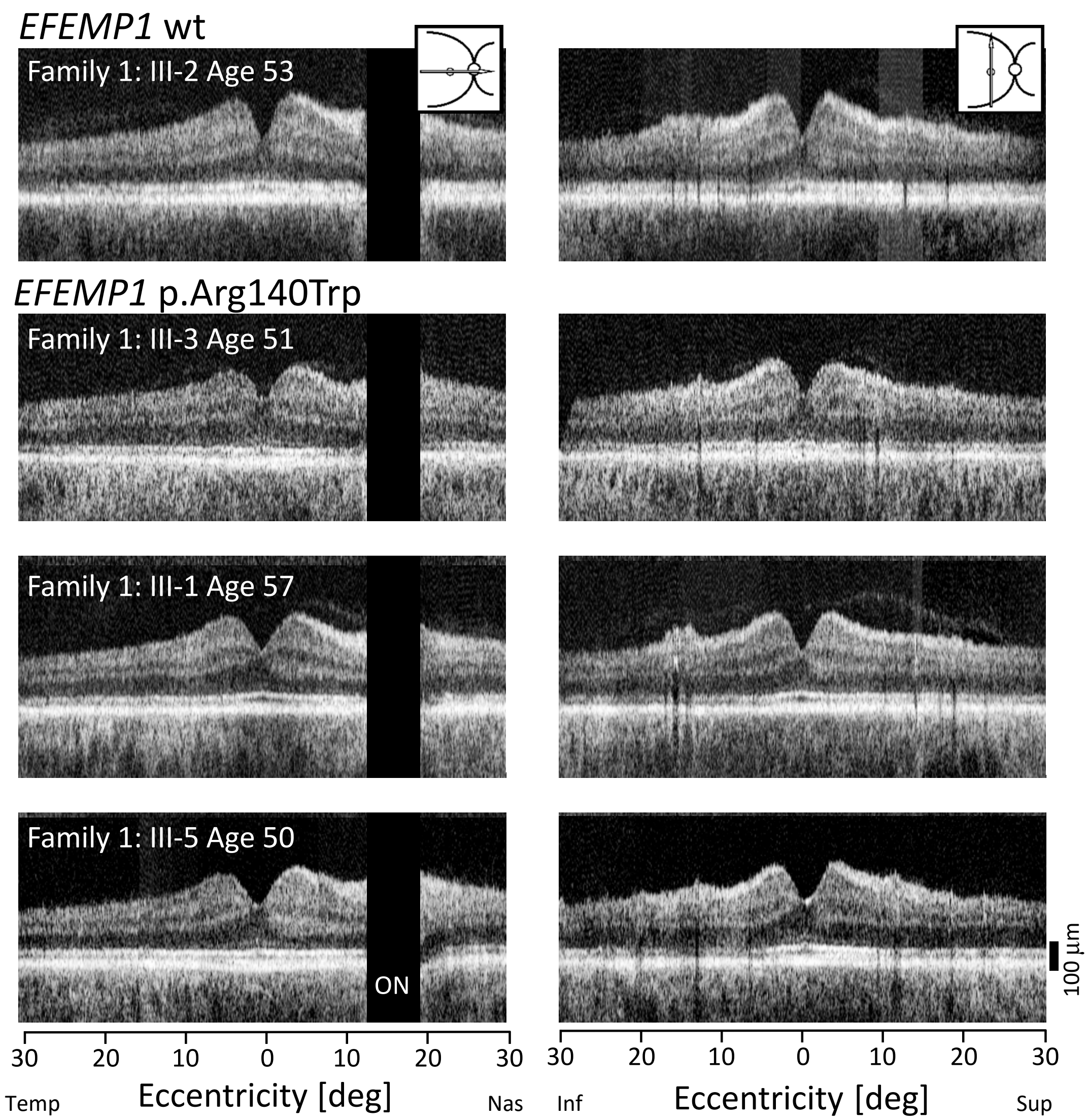

© 2026 Stanton CM et al. *JAMA Ophthalmology.*

eFigure 7. In silico Dimer-Interface Modeling of EFEMP1 p.Arg140Trp

In silico dimer-interface modeling (AlphaFold 3 / Rosetta) predicts that residue 140 can localize to a head-to-tail subunit interface adjacent to aromatic residues Tyr419 and Phe476 on the opposing chain. Substitution of Arg140 by Trp is predicted to stabilize this interface via alternative favorable aromatic packing interactions, including edge-to-face and offset face-to-face configurations,[1] with negative ΔΔG estimates for complex formation. Ring-to-ring and ring-to-atom distances are displayed in ångströms.

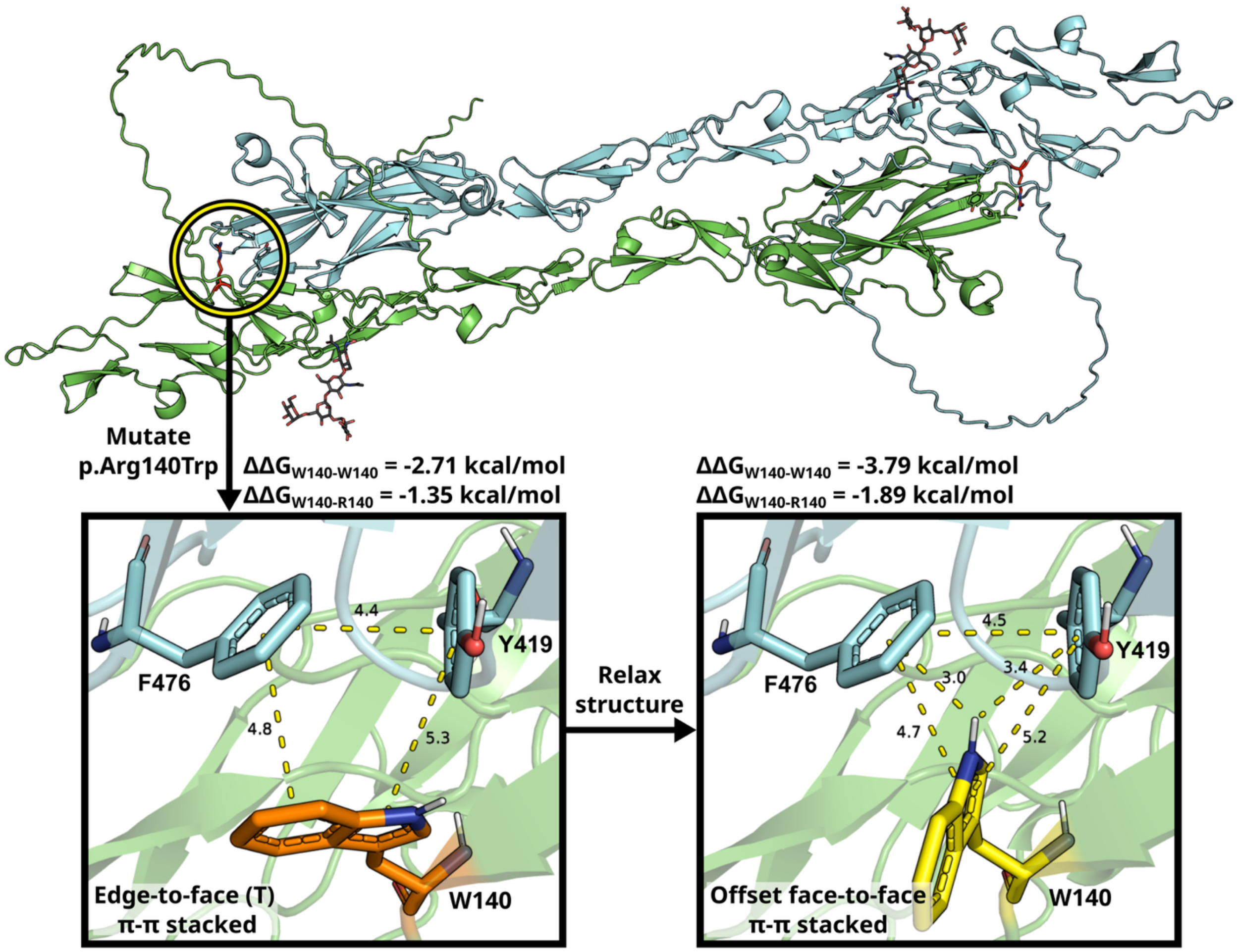

© 2026 Stanton CM et al. *JAMA Ophthalmology*.

eDiscussion

EFEMP1 p.Arg140Trp is less well characterized and has been reported to be indistinguishable from wild-type in secretion/accumulation assays[14] or, as we and others have observed, to accumulate intracellularly and form intracellular dimers.[15,16] Secreted wild-type EFEMP1 exists as a non-covalent dimer under native conditions but has proved challenging to visualize following SDS-PAGE, even under non-reducing conditions unless cysteine mutations have been introduced.[17,18] In our overexpression system, p.Arg140Trp showed increased intracellular dimer and higher-order oligomers under non-reducing conditions (Figure 4B), consistent with abnormal quaternary assembly. This is compatible with previous work using a similar experimental system.[15] Our observations extend prior reports that EFEMP1 dimers are detectable under native conditions but are difficult to preserve after SDS-PAGE, and they support the possibility that p.Arg140Trp alters folding or cysteine accessibility at the N-terminus of EFEMP1 in a manner that promotes aberrant intermolecular interactions. Notably, some p.Arg140Trp protein was secreted by transiently transfected HEK293T cells in this study, corresponding to approximately 20% of the total wild-type EFEMP1 secreted. When secreted EFEMP1 was examined under non-reducing conditions, both variant forms of EFEMP1 and wild-type protein could be visualized as a monomer. This contrasts to a spectrum of Cys variants that have been investigated in vitro, where abnormal disulfide bonds stabilize dimers, suggesting that non-covalent intermolecular bonding stabilizes p.Arg140Trp quaternary structure. In silico interface modeling (AlphaFold3/Rosetta) further supports this interpretation by identifying a plausible subunit interface in which Trp140 can significantly stabilize dimer packing near Tyr419/Phe476 compared to wild-type EFEMP1, thus impairing secretion.

Additional work is required to validate the impact of p.Arg140Trp when co-expressed with wild-type EFEMP1 to recapitulate the heterozygous genotype of individuals in this study. Our in silico modelling suggests that the gain-of-function multimerization would be less energetically favorable for a dimer formed from one wild-type and one mutant monomer than for a p.Arg140Trp dimer. This suggests that in an intracellular pool containing equally expressed wild-type and p.Arg140Trp EFEMP1, intracellular multimerization, and thus, impaired secretion, may be less pronounced. Alternatively, differences in retinal phenotype apparent between these two variants in EFEMP1 may reflect differences in extracellular aggregation or different functional impacts of the variants upon EFEMP1 function in the extracellular space. The p.Arg345Trp form of EFEMP1 is detected in DHRD/ML deposits, binds TIMP-3, inhibits MMP2 and reduces ECM turnover, triggers local complement activation and alters proteoglycan content of the Bruch's membrane.[19–22] These remain to be tested for the p.Arg140Trp variant, but it is plausible that a variant affecting the atypical N-terminal calcium-binding EGF domain in EFEMP1 may alter protein-protein interactions in a different way to the p.Arg345Trp alteration in the sixth EGF domain. The N- and C-terminal regions of the protein adopt distinct conformations when examined by rotary shadowing followed by electron microscopy, as predicted by AlphaFold.[23]

© 2026 Stanton CM et al. *JAMA Ophthalmology*.

eReferences

1. Calinsky R, Levy Y. Aromatic Residues in Proteins: Re-Evaluating the Geometry and Energetics of π–π, Cation−π, and CH−π Interactions. *J Phys Chem B*. 2024;128(36):8687-8700. doi:10.1021/acs.jpcb.4c04774

2. Thormann A, Halachev M, McLaren W, et al. Flexible and scalable diagnostic filtering of genomic variants using G2P with Ensembl VEP. *Nat Commun*. 2019;10(1):2373. doi:10.1038/s41467-019-10016-3

3. Gene2Phenotype (G2P) Eye disorders panel (Eye). Accessed February 13, 2026. https://www.ebi.ac.uk/gene2phenotype/panel/Eye

4. McLaren W, Gil L, Hunt SE, et al. The Ensembl Variant Effect Predictor. *Genome Biol*. 2016;17(1):122. doi:10.1186/s13059-016-0974-4

5. Stanton CM, Borooah S, Drake C, et al. Novel pathogenic mutations in C1QTNF5 support a dominant negative disease mechanism in late-onset retinal degeneration. *Sci Rep*. 2017;7(1):12147. doi:10.1038/s41598-017-11898-3

6. Milam AH, Curcio CA, Cideciyan A V, et al. Dominant late-onset retinal degeneration with regional variation of sub-retinal pigment epithelium deposits, retinal function, and photoreceptor degeneration. *Ophthalmology*. 2000;107(12):2256-2266. doi:10.1016/s0161-6420(00)00419-x

7. Abramson J, Adler J, Dunger J, et al. Accurate structure prediction of biomolecular interactions with AlphaFold 3. *Nature*. 2024;630(8016):493-500. doi:10.1038/s41586-024-07487-w

8. Hulleman JD, Kelly JW. Genetic ablation of N-linked glycosylation reveals two key folding pathways for R345W fibulin-3, a secreted protein associated with retinal degeneration. *The FASEB Journal*. 2015;29(2):565-575. doi:10.1096/fj.14-255414

9. Bateman A, Martin MJ, Orchard S, et al. UniProt: the Universal Protein Knowledgebase in 2025. *Nucleic Acids Res*. 2025;53(D1):D609-D617. doi:10.1093/nar/gkae1010

10. Huang C, Moremen KW. Editor's Choice JAAG: a JSON input file assembler for AlphaFold 3 with glycan integration. *Glycobiology*. 2025;36(1). doi:10.1093/glycob/cwaf083

11. Alford RF, Leaver-Fay A, Jeliazkov JR, et al. The Rosetta All-Atom Energy Function for Macromolecular Modeling and Design. *J Chem Theory Comput*. 2017;13(6):3031-3048. doi:10.1021/acs.jctc.7b00125

12. Park H, Bradley P, Greisen P, et al. Simultaneous Optimization of Biomolecular Energy Functions on Features from Small Molecules and Macromolecules. *J Chem Theory Comput*. 2016;12(12):6201-6212. doi:10.1021/acs.jctc.6b00819

13. Frenz B, Lewis SM, King I, DiMaio F, Park H, Song Y. Prediction of Protein Mutational Free Energy: Benchmark and Sampling Improvements Increase Classification Accuracy. *Front Bioeng Biotechnol*. 2020;8. doi:10.3389/fbioe.2020.558247

14. Woodard DR, Nakahara E, Hulleman JD. Clinically-identified C-terminal mutations in fibulin-3 are prone to misfolding and destabilization. *Sci Rep*. 2021;11(1):2998. doi:10.1038/s41598-020-79570-x

15. Mackay DS, Bennett TM, Shiels A. Exome Sequencing Identifies a Missense Variant in EFEMP1 Co-Segregating in a Family with Autosomal Dominant Primary Open-Angle Glaucoma. *PLoS One*. 2015;10(7):e0132529. doi:10.1371/journal.pone.0132529

16. Collantes ERA, Delfin MS, Fan B, et al. EFEMP1 rare variants cause familial juvenile-onset open-angle glaucoma. *Hum Mutat*. 2022;43(2):240-252. doi:10.1002/humu.24320

17. Collier GE, Roovers AL, Hulleman JD. A spectrum of cysteine mutations in fibulin-3 highlight its disulfide bonding complexity and potential to induce ER stress. *Sci Rep*. 2025;15(1):30028. doi:10.1038/s41598-025-14561-4

© 2026 Stanton CM et al. *JAMA Ophthalmology.*

18. Djokic J, Fagotto-Kaufmann C, Bartels R, Nelea V, Reinhardt DP. Fibulin-3, -4, and -5 Are Highly Susceptible to Proteolysis, Interact with Cells and Heparin, and Form Multimers. *Journal of Biological Chemistry*. 2013;288(31):22821-22835. doi:10.1074/jbc.M112.439158

19. Marmorstein LY, Munier FL, Arsenijevic Y, et al. Aberrant accumulation of EFEMP1 underlies drusen formation in Malattia Leventinese and age-related macular degeneration. *Proc Natl Acad Sci U S A*. 2002;99(20):13067-13072. doi:10.1073/pnas.202491599

20. Klenotic PA, Munier FL, Marmorstein LY, Anand-Apte B. Tissue Inhibitor of Metalloproteinases-3 (TIMP-3) Is a Binding Partner of Epithelial Growth Factor-containing Fibulin-like Extracellular Matrix Protein 1 (EFEMP1). *Journal of Biological Chemistry*. 2004;279(29):30469-30473. doi:10.1074/jbc.M403026200

21. Fernandez-Godino R, Garland DL, Pierce EA. A local complement response by RPE causes early-stage macular degeneration. *Hum Mol Genet*. 2015;24(19):5555-5569. doi:10.1093/hmg/ddv287

22. Zayas-Santiago A, Cross SD, Stanton JB, Marmorstein AD, Marmorstein LY. Mutant Fibulin-3 Causes Proteoglycan Accumulation and Impaired Diffusion Across Bruch's Membrane. *Invest Ophthalmol Vis Sci*. 2017;58(7):3046. doi:10.1167/iovs.17-21720

23. Zhang Y, Marmorstein LY. Focus on molecules: Fibulin-3 (EFEMP1). *Exp Eye Res*. 2010;90(3):374-375. doi:10.1016/j.exer.2009.09.018

© 2026 Stanton CM et al. *JAMA Ophthalmology*.
