# Supplementary material for: Widening the Spectrum of Disease Expression due to Heterozygous Variants in EFEMP1
Source: JAMA Ophthalmol. 2026 Sep 10;144(10):915–24. doi: 10.1001/jamaophthalmol.2026.3828 (PMC13563294; doi:10.1001/jamaophthalmol.2026.3828)
Supplement: Supplementary file 2 [file jamaophthalmol-e263828-s002.pdf]

Data Sharing Statement

Stanton. Widening the Spectrum of Disease Expression due to Heterozygous Variants in EFEMP1. *JAMA Ophthalmol*. Published September 10, 2026.
doi:10.1001/jamaophthalmol.2026.3828

Data
Data available: No

Additional Information
Explanation for why data not available: Granular patient-level data provided as part of the manuscript.
